# Chemistry of Lipoquinones: Properties, Synthesis, and Membrane Location of Ubiquinones, Plastoquinones, and Menaquinones

**DOI:** 10.3390/ijms232112856

**Published:** 2022-10-25

**Authors:** Margaret M. Braasch-Turi, Jordan T. Koehn, Debbie C. Crans

**Affiliations:** 1 Chemistry Department, Colorado State University, Fort Collins, CO 80523, USA; 2 Cell & Molecular Biology Program, Colorado State University, Fort Collins, CO 80523, USA

**Keywords:** lipoquinone, ubiquinone, plastoquinone, menaquinone, electron transport chain, hydrophobic, electrochemistry, solubility, conformation, membrane location

## Abstract

Lipoquinones are the topic of this review and are a class of hydrophobic lipid molecules with key biological functions that are linked to their structure, properties, and location within a biological membrane. Ubiquinones, plastoquinones, and menaquinones vary regarding their quinone headgroup, isoprenoid sidechain, properties, and biological functions, including the shuttling of electrons between membrane-bound protein complexes within the electron transport chain. Lipoquinones are highly hydrophobic molecules that are soluble in organic solvents and insoluble in aqueous solution, causing obstacles in water-based assays that measure their chemical properties, enzyme activities and effects on cell growth. Little is known about the location and ultimately movement of lipoquinones in the membrane, and these properties are topics described in this review. Computational studies are particularly abundant in the recent years in this area, and there is far less experimental evidence to verify the often conflicting interpretations and conclusions that result from computational studies of very different membrane model systems. Some recent experimental studies have described using truncated lipoquinone derivatives, such as ubiquinone-2 (UQ-2) and menaquinone-2 (MK-2), to investigate their conformation, their location in the membrane, and their biological function. Truncated lipoquinone derivatives are soluble in water-based assays, and hence can serve as excellent analogs for study even though they are more mobile in the membrane than the longer chain counterparts. In this review, we will discuss the properties, location in the membrane, and syntheses of three main classes of lipoquinones including truncated derivatives. Our goal is to highlight the importance of bridging the gap between experimental and computational methods and to incorporate properties-focused considerations when proposing future studies relating to the function of lipoquinones in membranes.

## 1. Introduction

Lipoquinones are molecules involved in a variety of biological processes. Their appearance may be simple, but their properties are deceptively complex. This class of lipids contains a redox active, polar 1,4-quinone headgroup and a hydrophobic sidechain consisting of several isoprenoid groups. Lipoquinones are components within the highly ordered lipid bilayer [1,2,3]. One example of lipoquinone function is to act as the electron carriers responsible for transferring electrons between membrane-bound protein complexes within the electron transport chain, ultimately producing adenosine triphosphate (ATP). The structure of lipoquinones, such as headgroup structure and composition of the sidechain, varies between whether the biological system is bacterial, plant, or mammalian and their biological functions. This is not surprising because the structure of the quinone headgroup and isoprenoid sidechain has been used for taxonomic identification of prokaryotes and eukaryotes prior to the development of genetic classification [4,5].

With this in mind, it is not fully understood how the length and degree of unsaturation along the lipoquinone sidechain impacts both their chemical and biological properties other than the fact that lipoquinones are required for survival [5,6,7]. The relationship between structural diversity and chemical properties and the biological function of lipoquinones is a question that is often overlooked or ignored. However, Hihi and coworkers explored the significance of the sidechain length through a ubiquinone (UQ) -derivative feeding study with *Caenorhabditis elegans clk-1* (*C. elegans*) mutants [6]. Therein, UQ-6 to UQ-9 were fed to UQ-8-depleted *C. elegans* to determine their effects on growth and behavior. They found the longer chain derivatives, like UQ-9 and UQ-8, restored the phenotypic behavior. The shorter chain derivatives, UQ-7 and UQ-6 sustained continuous growth in the mutants but halted certain UQ-dependent processes, such as fertility, and slowed development and behavior of *C. elegans* [6]. This study suggests the sidechain length plays a significant role in the development of this model organism, but it does not address the properties of each UQ-derivative and how it could affect the results.

A study performed by Okada and coworkers used *Saccharomyces cerevisiae* (*S. cerevisiae*) as a model organism to assess the biological significance of the sidechain length of ubiquinone within the organism [7]. Different types of prenyl diphosphate synthases were expressed to produce UQ-5 to UQ-10, and the growth rate of each mutant was evaluated. UQ-6 is the preferred lipoquinone of *S. cerevisiae* showing the highest growth rate. The other UQ-derivatives did not affect the growth rate significantly which suggests the headgroup is more important that the sidechain. In their conclusion, they suggest the length of the sidechain may play an important role in adjusting the hydrophobicity of the UQ-derivative to the membrane environment [7]. Together these studies support the significance the length of the sidechain, but both clearly indicate the headgroup is the critical moiety for survival.

**Figure 1:**
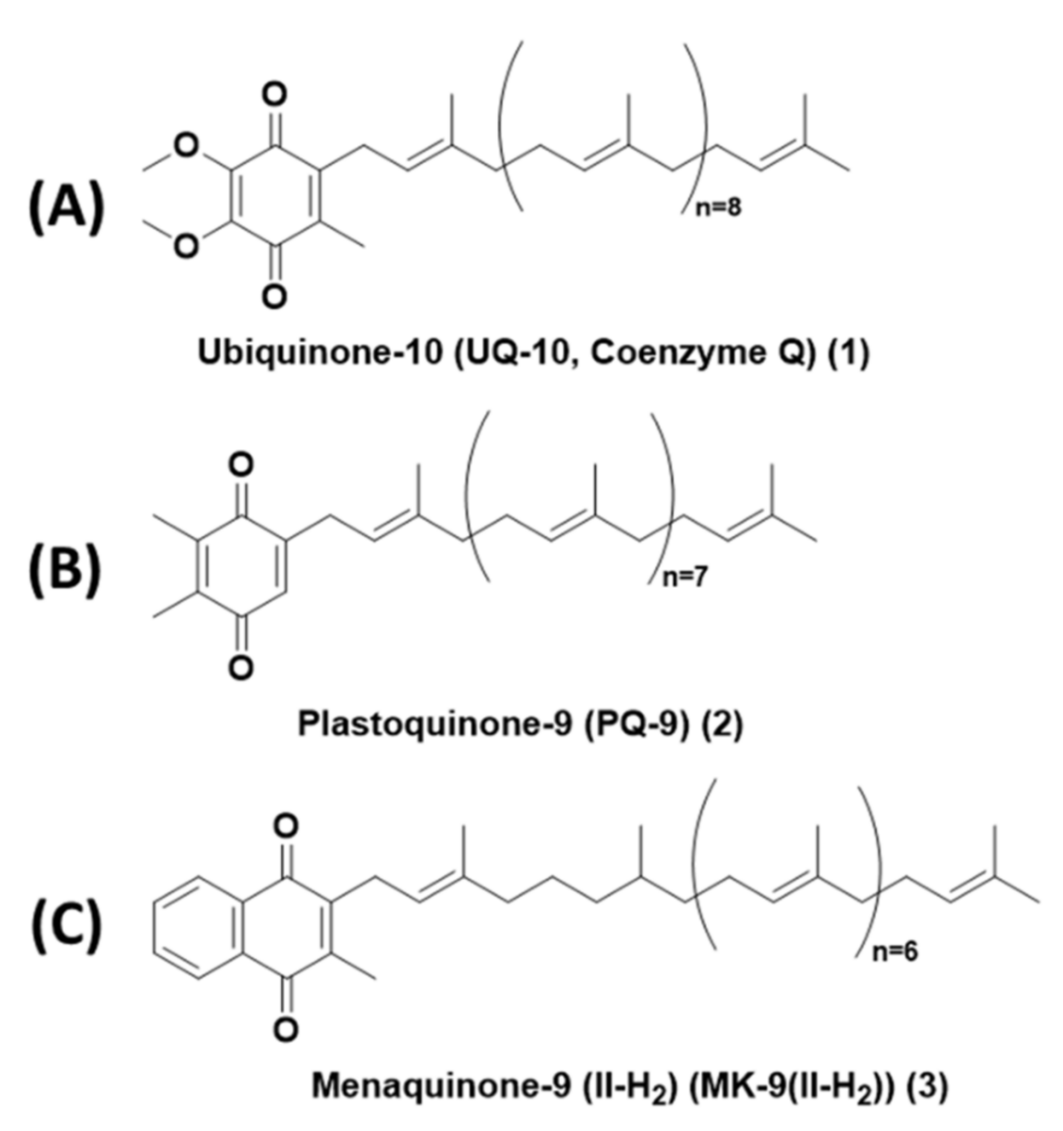
Structures of representative lipoquinones from each subclass. (**A**) Ubiquinone-10, also known as Coenzyme Q10 or UQ-10. (**B**) Plastoquinone-9 (PQ-9). (**C**) Menaquinone-9(II-H_2_) (MK-9(II-H_2_)).

In the present review, we describe the chemical properties of lipoquinones such as hydrophobicity, solubility, conformation, and redox potential organized according to the quinone headgroup type. We will focus on three subclasses of lipoquinones: ubiquinone, plastoquinone, and menaquinone, which differ by headgroup structure. Ubiquinones (UQ) are comprised of a 1,4-benzoquinone headgroup with four substituents: two methoxy groups, isoprenyl sidechain, and one methyl group adjacent to the sidechain. Ubiquinones are found within the inner mitochondrial membrane, where they facilitate the electron transport chain of eukaryotes. Ubiquinone-10, also known as UQ-10 or coenzyme Q10, is the most well-known lipoquinone and UQ-derivative (Figure 1A). Plastoquinones (PQ) are also comprised of a 1,4-benzoquinone headgroup, but differs from ubiquinones with two methyl group substituents, as seen in plastoquinone-9 (PQ-9, Figure 1B). Plastoquinones are found in the thylakoid membrane of chloroplasts and facilitate photophosphorylation. Menaquinones (MK) are comprised of a 1,4-naphthoquinone headgroup with a methyl group adjacent to the sidechain, as seen in menaquinone-9(II-H_2_) (MK-9(II-H_2_), Figure 1C). Menaquinones are found in the cytoplasmic membrane electron transport chain of prokaryotes.

For all three subclasses, the isoprenoid sidechains can vary in the length and degree of unsaturation. Some species have been found to have saturated isoprene units along the sidechain. The isoprenoid sidechains are biosynthesized from isopentyl diphosphate and dimethylallyl diphosphate via one of three pathways: mevalonate pathway, 2-C-methyl-D-erythritol 4-phosphate, and 1-deoxy-D-xylulose 5-phosphate pathway [8]. The origins of these pathways are hypothesized to have emerged from a common ancestor via the mevalonate pathway and diverged throughout evolution to the other pathways [9,10].

The 1,4-quinone headgroup is responsible for the redox activity of lipoquinones. If all lipoquinones are capable of transferring electrons, it is curious that nature evolved such structural diversity in headgroups across lipoquinones to perform the same operations. The chemical redox properties of the headgroups vary and play a role in the setting the baseline reduction potentials required to transfer electrons between membrane-bound protein complexes. This trend appears true from an evolutionary perspective. Of the three lipoquinones discussed in this review, menaquinones evolved first within prokaryotes, which includes bacteria and archaea, which existed within a mostly anerobic environment and thus underwent anaerobic respiration. As time progressed, the environment became saturated with harsh UV light from the sun, which undoubtedly caused damage to cells. At some point, bacteria within the cyanobacteria phylum evolved plastoquinones to harvest energy from light to generate ATP via photophosphorylation that ultimately leads to the release of oxygen into the atmosphere. Interestingly, certain classes of cyanobacteria use both plastoquinone and menaquinone derivatives to facilitate photosynthesis. PQ-9 is the electron transfer agent for photosystem II, and phylloquinone (MK-4 (II, III, IV-H_6_) or vitamin K_1_) is the electron transfer agent for photosystem I [11,12]. As more oxygen was introduced to the atmosphere, organisms needed to adapt lipoquinones that could withstand the oxidizing atmosphere. Eventually, organisms in the pseudomonadota (previously known as proteobacteria) phylum evolved ubiquinones because they are more resistant to oxidation than menaquinones (see the discussion in Section 2.3) [13,14,15,16]. This change in headgroup allowed for aerobic respiration to occur. Certain classes within the phylum, specifically α-, β-, and γ-proteobacteria, use both menaquinones and ubiquinones for electron transport. For example, *Escherichia coli* (*E. coli*), a γ-proteobacterium, can switch between lipoquinones depending on specific growth conditions. Ubiquinone is best suited for aerobic growth conditions, and menaquinone is used in anaerobic growth conditions [15]. More than one type of lipoquinone in one organism supports the concept that the properties of lipoquinones are tailored to their redox activity.

Eukaryotic organisms primarily utilize ubiquinones for oxidative phosphorylation; however, all members of the plantae kingdom contain both ubiquinones and plastoquinones. The incorporation of each lipoquinone within this domain are hypothesized to have originated from the occurrence of two endosymbiotic relationships between a eukaryotic ancestor and organisms in both pseudomonadota and cyanobacteria phyla. Since all eukaryotes contain ubiquinone, the endosymbiosis of an α-, β-, or γ-proteobacterium must have occurred first. Over time, the endosymbiont became the mitochondria, an organelle that houses the electron transport chain in eukaryotes [17,18,19,20]. Similarly, the origin of chloroplast organelles is thought to have occurred via the endosymbiosis of a cyanobacterium into a eukaryotic organism [21]. The incorporation of photosynthetic machinery into the cells allows these organisms to use plastoquinones to harness the energy from light for photosynthesis and use ubiquinones for oxidative phosphorylation within the mitochondria [17]. The emergence of different lipoquinones with their intrinsic properties allowed for organisms to thrive in environments of varying levels of hostility.

The isoprenoid sidechain is thought to anchor the lipoquinone into the lipid bilayer. The sidechain is comprised of both single and double bonds that provide rigidity (double bond) and flexibility (single bonds) via multiple degrees of rotational freedom. The length and degree of unsaturation along the sidechain vary between species. For example, UQ-10 is the most common lipoquinone in humans, and UQ-9 is the prevalent species in rodents [22]. In prokaryotes, there is greater sidechain diversity of menaquinones between species. For example, *Halococcus morrhuae* contains MK-8(VIII-H_2_), whereas *Mycolicibacterium smegmatis* (previously categorized as *Mycobacterium smegmatis, M. smeg)* and *Corynebacterium tuberculostearicum* contain MK-9 (II-H_2_) and MK-9, respectively [4]. The only difference between these two MK-derivatives is the presence of a saturated (reduction of a specific double bond) isoprene unit along the sidechain. In addition to the structure of the headgroup, electrochemical studies have shown the composition of the sidechain can affect the reduction potential of lipoquinones [23]. For example, the redox potentials of MK-4 and MK-8 have been found to be −20 to −30 mV and −80 mV, respectively. The variance in redox potentials shows the length of the sidechain impacts the reduction potential of the lipoquinone. Additionally, the reduction potential of MK-4 and phylloquinone (vitamin K_1_ or MK-4 (II, II, IV- H_6_)) are −20 to −30 mV and −500 to −700 mV, respectively. Even though MK-4 and phylloquinone contain the same number of carbons in the sidechain, the presence of three saturated isoprene units significantly decreases the reduction potential [23]. Therefore, modifications to the sidechain are very likely to play a role in fine tuning the reduction potentials within their respective protein complexes.

In addition to lipoquinone’s role in the electron transport chain, they have antioxidant properties [5], which protects against lipid peroxidation and the damaging effects of reactive oxygen species throughout the membrane [11]. Menaquinones are essential for human health as members of the vitamin K family of compounds: vitamin K_1_ (phylloquinone), vitamin K_2_ (MK-4), and vitamin K_3_ (menadione, which lacks a sidechain). These vitamins critical for mammalian metabolism and must be supplied through food or obtained from symbiotic bacteria living within the gut [24]. Vitamin K derivatives, especially vitamin K_1_, are cofactors for blood clotting [25,26], components for modifications of proteins through post-translational processes [24], and regulators of bone metabolism and growth [26]. A review detailing recent advances in the roles of vitamin K has recently been reported [27]. UQ-10 has been found to behave as a redox sensor for the regulation of gene expression [28]. PQ-9 serves as a redox sensor for the regulation of carotenoid biosynthesis [29,30] and physiological responses to changes in the quality and intensity of light [31].

**Figure 2:**
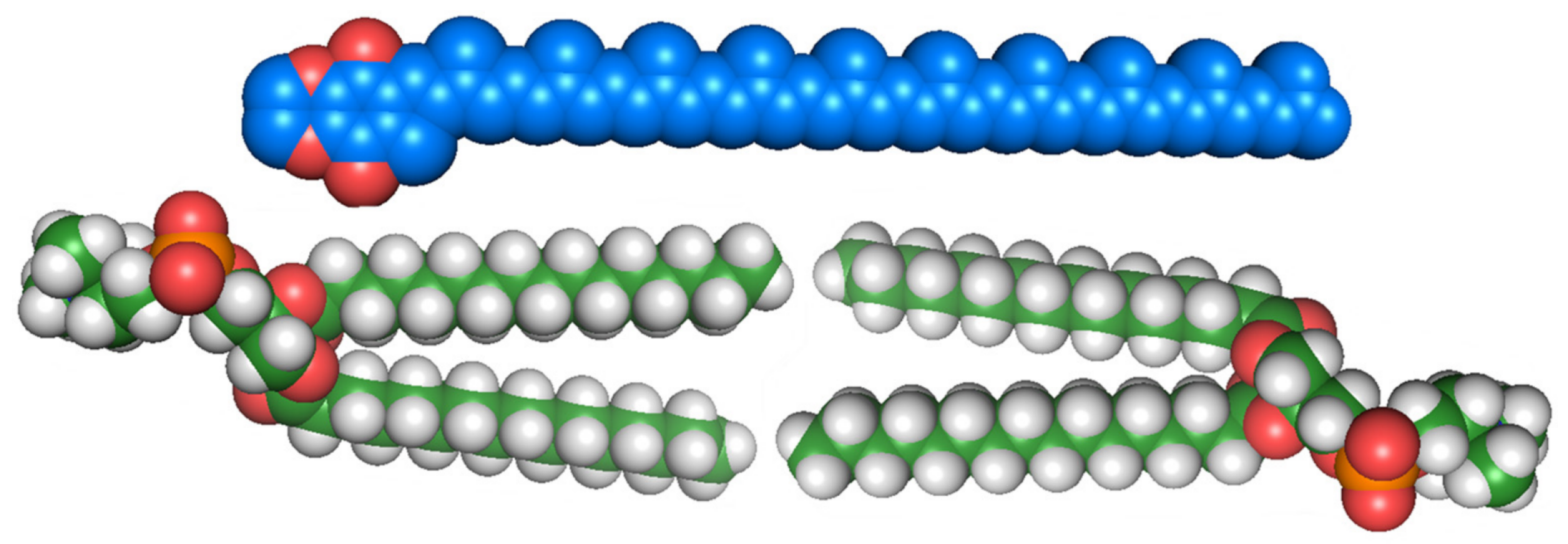
Space-filling model of UQ-10 (C_59_H_90_O_4_) (top) and model bilayer of dipalmitoylphosphatidylcholine (DPPC) (16:0) (bottom). Illustration was inspired by a figure that appeared in Ref. [32].

Much of the lipoquinone literature has focused on their redox activity, interactions as cofactors, and contributions to healthy development of biological systems. However, little is known about their properties within the membrane when they are not associated with proteins. Diagrams depicting the electron transport chain generally show their location and function in the membrane as an amorphous shape across the whole lipid bilayer and labeled simply “Q”. This oversimplification perpetuates the disconnect between the location of lipoquinones and their properties. Early investigations into the location of lipoquinones began by considering their size compared to the size of the membrane. The extended length of UQ-10 is approximately 56 Å, which could penetrate and span both leaflets of a typical phospholipid bilayer, including both hydrophilic and hydrophobic regions of the membrane (see Figure 2) [32]. Therefore, UQ-10 is too long to be accommodated within the membrane unless the isoprenoid chain is folded in some fashion or located parallel to the membrane surface at the midplane. A handful of studies have been performed to determine the location of the lipoquinone headgroup in the membrane; however, the topic remains controversial as no clear consensus has emerged. A summary of these studies’ headgroup locations can be found in Table 1 [1,33,34,35,36,37,38,39,40,41,42,43,44,45,46,47,48,49,50,51,52,53,54,55,56,57,58,59,60].

**Figure 3:**
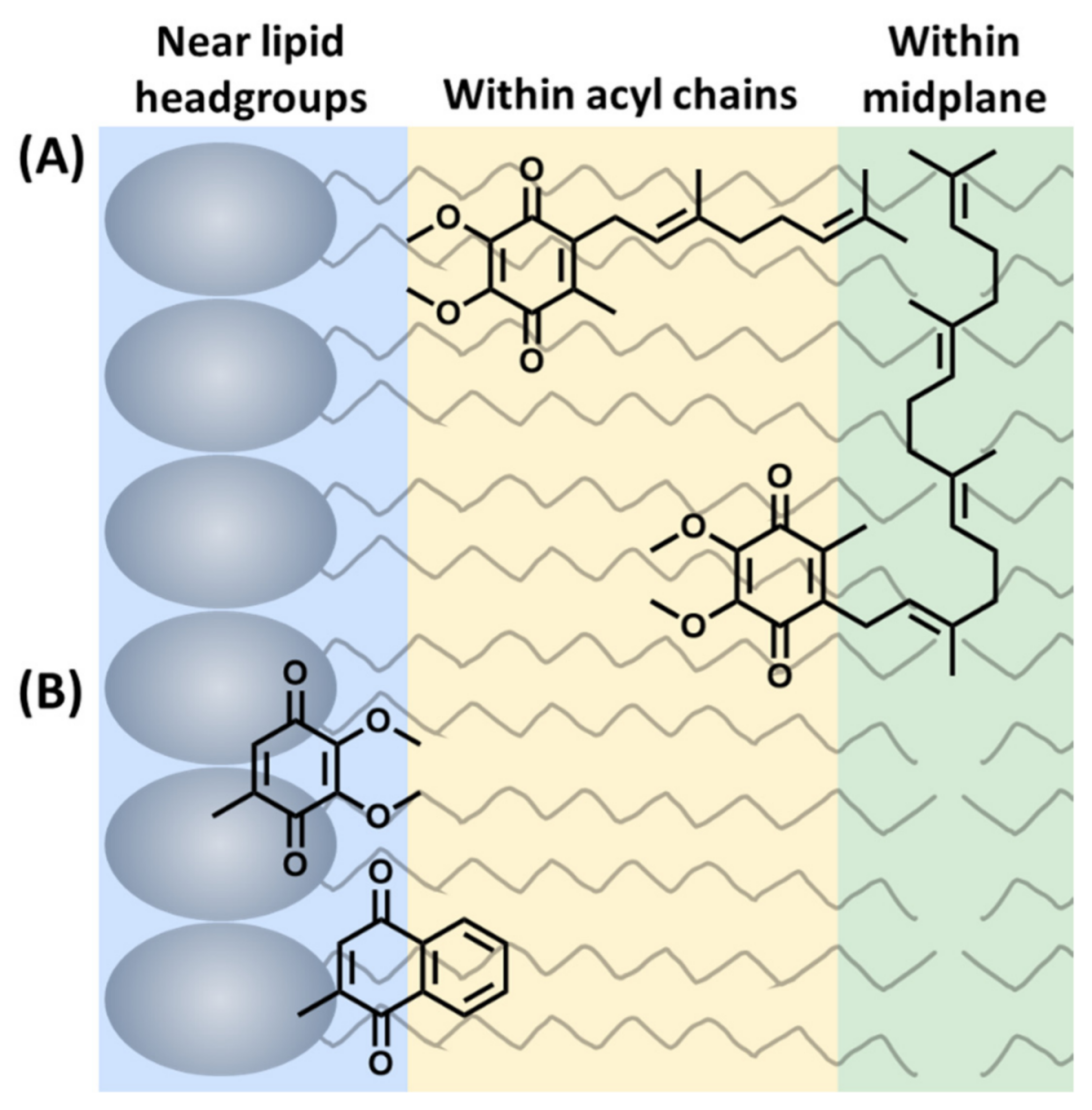
Three possible locations of the lipoquinone headgroup: (**A**) Example of the influence of sidechain on the location of the headgroup. UQ-2 and UQ-4 are both shown as illustrative examples within the acyl chains or within the midplane. (**B**) Example of the influence of headgroup polarity on the location of the quinone headgroup in the membrane (sidechains omitted for clarity). This figure was originally published in Ref. [41] to include UQ-derivatives in (**A**). It is licensed under CC-BY 4.0.

The literature places the quinone headgroup in three locations across the bilayer: (1) near the phospholipid headgroup or the interface, (2) within the acyl chains, and (3) within the midplane (Figure 3). From these studies, ubiquinones were most often determined to be near the lipid headgroups, plastoquinones were most often determined to be within the bilayer midplane, and menaquinones were most often determined to at or near the interface (Figure 3). However, there were numerous studies favoring other locations. Although these results seem correlated to the polarity of the headgroup, analogous methods, such as NMR studies with chiral shift agents, returned conflicting results. A summary of the materials and methods is described below in Section 3.1. Undoubtedly, a more in-depth consideration of how the properties and structural diversity of lipoquinones would assist the interpretation of the results of experimental and computational studies.

In addition to the controversy surrounding the location of lipoquinones in the membrane, there is also considerable dispute over what influences the position of the quinone headgroup in the membrane (Figure 3). Many studies suggest the length of the isoprene sidechain determines the location of the headgroup where the longer derivatives are found closer to the midplane. For example, based on the trends observed for UQ-derivatives [33,34,35,49], UQ-4 would be expected to be closer to the midplane than UQ-2 (Figure 3A). This is thought to be a result of the longer sidechain embedding deep into the midplane due to the hydrophobic effect. On the other hand, a few recent studies have shown the type of headgroup is the major influence on the position of lipoquinones in the membrane (Figure 3B) [1,41]. For example, the ubiquinone headgroup is more polar than menaquinone; therefore, it would be expected that ubiquinone would be closer to the interface than menaquinone. The truth likely has several different cases depending on the biological system, and too few experimental studies have been reported to provide a consistent picture. In addition, some computational studies have shown the location of the lipoquinone headgroup is dependent on its proximity to proteins. In a recent computational study, the dominant species in lipid-rich environments was found to be the “swimming” quinone found near the interface, and “diving” quinones were the dominant species in protein-rich environments found near the midplane [2]. However, it is likely that some of these quinones are moving within the membrane, making the studies even more difficult. Therefore, it is important to consider what additional components are present in the membrane when interpreting the results of computational and experimental studies. For this review, we will primarily focus on the chemical and physical properties of lipoquinones, and in organic solvents and lipid environments. We will discuss how these properties (i.e., hydrophobicity, Section 2.1; solubility, Section 2.2; electrochemical properties, Section 2.3; conformation of lipoquinones, Section 2.4 and the three lipoquinones, Section 2.4) could impact the properties of the lipoquinone and be a cause of the inconsistent observations reported in experimental and computational studies (summarized below in Section 3.1).

In the following review, we will describe the chemistry of lipoquinone with the objective to begin to consider: (i) how the properties of lipoquinones affect their shape, location, and redox activity, (ii) how their chemical environments impact the chemical and physical properties, and (iii) how their properties affect their biological functions within the membrane.

## 2. Properties of Lipoquinones

Lipoquinones are considered hydrophobic, but the consequences of this property are often not taken into consideration when approaching experiments and interpreting data. Herein we will discuss the hydrophobicity, solubility, electrochemistry, and conformational properties of lipoquinones.

### 2.1. Hydrophobicity

Although the presence of both polar and nonpolar regions of the molecules makes lipoquinones amphipathic, the molecules are overall described as hydrophobic. To allow for comparison of these molecules, we compiled reported logP values from the literature and PubChem and calculated logP values of the lipoquinones with different headgroups, sidechain lengths and redox states in Table 2 [61,62,63,64,65]. The clogP values were calculated using programs such as Molinspiration and Chemicalize [66,67]. From the data in Table 2, it is clear the length of the sidechain influences logP and the value of logP increases as the length of the sidechain increases. For example, the logP values for UQ-0, UQ-2, and UQ-10 were found to be 0.8, 4.6, and 19.4, respectively [65]. The 20-fold change in logP demonstrates the significant effect an additional 40 carbons has on the hydrophobic properties of lipoquinones.

Although the headgroup is the polar part of the lipoquinone, it also contributes to the hydrophobicity. The logP values for UQ-0, PQ-0, and MK-0 were found to be 0.8, 1.2, and 2.2, respectively [65]. The hydrophobicity of the headgroup is often overlooked even though the numeric changes are less dramatic than obtained from different sidechains lengths. Recognizing that the headgroup is hydrophobic is particularly important when interpreting results.

It is logical that UQ-0 has the lowest logP value (0.8) because it is the most polar headgroup, and it has two additional polar C-O bonds. PQ-0 (1.2) and MK-0 (2.2) are more nonpolar, but the addition of the extra ring in the naphthoquinone headgroup plays a significant role in increasing the logP of MK-0. Another important trend to note is how the reduced forms, the corresponding quinols, affects the logP values. Surprisingly, nearly all the lipoquinols have a slightly higher logP value than their respective quinones [65,66,67]. For example, MK-9 and MKH_2_-9 were found to have logP values of 18.2 and 19.6, respectively [65]. The increase in logP seems contradictory when considering the formation of the hydroxy groups, which allows for the introduction of two additional hydrogen bonds, thus enabling the quinols to interact with other polar molecules. This, however, is not the case, which suggests that hydrogen bonds are not very significant. The increase in logP of the quinols could be due to the formation of the aromatic ring upon reduction; therefore, increasing the electron density on the aromatic ring. The two hydroxy groups on the aromatic ring are more likely to donate their electron density to the ring and thus not available for hydrogen bonding. In addition, the aromatic ring allows for additional noncovalent interactions, like π- π stacking, with the double bonds in the sidechain and the headgroups of other lipoquinones.

It is also important to note the variability in the calculated logP values of the same lipoquinone derivatives across Table 2. For example, UQ-10 was found to have a logP value of 19.4, 10.51, and 17.156 from PubChem, Molinspiration, and Chemicalize, respectively. Although all three values suggest these molecules are sufficiently hydrophobic, the differences in the numbers calculated by the different programs are significant. The discrepancies between sources could have major implications on how computational studies are performed and how the results are interpreted. For example, if a researcher used the PubChem value for UQ-10 (19.4) and another used the Molinspiration value (10.51) in the same experiments, such studies could result in conflicting conclusions for the same system.

The hydrophobicity of lipoquinones also plays a role in conformational structure. In terms of noncovalent interactions, how lipoquinones interact with their surrounding environment will be severely influenced by the hydrophobic effect. This feature led to their physical appearance to be described as “fat globs” which implies they do not adopt any specific conformations. However, polyisoprenoid molecules have been known to adopt specific conformations. For example, squalene epoxide was used in the biomimetic synthesis of cholesterol by Woodward and Bloch in 1953 [68]. Out of the 256 (2^8^) possible isomers, only one stereochemical outcome was achieved, consistent with squalene epoxide adopting a preorganized conformation prior to cyclization, and this preference for a single conformation affected the molecule’s properties and reactivity. Such conformational preferences are likely governed by the hydrophobic effect and intramolecular π- π stacking interactions and suggests that the conformational preferences of lipoquinones are likely to exist.

### 2.2. Solubility

Lipoquinones are found naturally in cell membranes as anticipated since they share amphipathic characteristics with the lipids in the bilayer. However, hydrophobic molecules are likely to exhibit solubility issues when added to aqueous solutions, and this limitation must be taken into consideration when designing experiments or analyses. For example, standard laboratory practices can affect the experimental outcome, such as use of plastic Eppendorf tubes a common container in biological studies. Since these opaque vessels obscure the contents, researchers are prevented from observing if a solute has completely dissolved. Therefore, it is entirely possible the researchers did not check to make sure the lipoquinone derivatives had completely dissolved.

Another example involves measurements of the electrochemical properties of lipoquinones because the results are dependent on whether the compounds are dissolved in organic, aqueous, or mixed organic-aqueous solutions/suspensions. When determining or using reduction potentials of biologically relevant molecules, such as lipoquinones, the insolubility in aqueous solution affects the reduction potentials that are measured in organic solvents. The corresponding reduction potentials in life sciences are extrapolated to aqueous-biological systems.

Surfactants or dimethyl sulfoxide are often used to solubilize hydrophobic compounds in aqueous media, but it comes at the risk of introducing contaminants or additives that are difficult to purify or cause changes in molecular properties of the target compounds in such complex mixtures. Solubility can therefore impact results obtained in biological assays, particularly with compounds such as lipoquinones. For example, MK-1 through MK-7 were investigated for their anticancer properties [64]. MK-1, MK-2, and MK-3 were found to exhibit cytotoxic activities, and the others, MK-4, MK-5, MK-6, and MK-7, did not [64]. The authors’ conclusion was that the longer chain derivatives were not cytotoxic and therefore inadequate candidates for further consideration in their study. An alternative interpretation of these result and explanation of the absence of cytotoxic activity for the longer derivatives is simply aqueous insolubility. Thus, the correlations and proposed patterns of the measured activities may not be based on the true cytotoxicity of these compounds, but was a consequence of their physical properties, which in this case was the poor water solubility of the hydrophobic MK-derivatives with sidechains longer than four isoprenoid groups.

In another study, the substrate affinity of MK-1, UQ-1, MK-4, and MK-9 for MenJ was investigated based on an enzyme assay using surfactant to solubilize the substrates tested [69]. In this study MenJ activity was observed when MK-1 and UQ-1 were used as substrates. However, as described above MK-4 and MK-9 are significantly less soluble, and the fact that no activity was observed in the enzyme assay with these two molecules (with one expected as the natural substrate) was attributed to the lack of solubility of MK-4 and MK-9. The lack of observed enzyme activity was because MK-4 and MK-9 are not soluble under the reported assay conditions [69].

**Figure 4:**
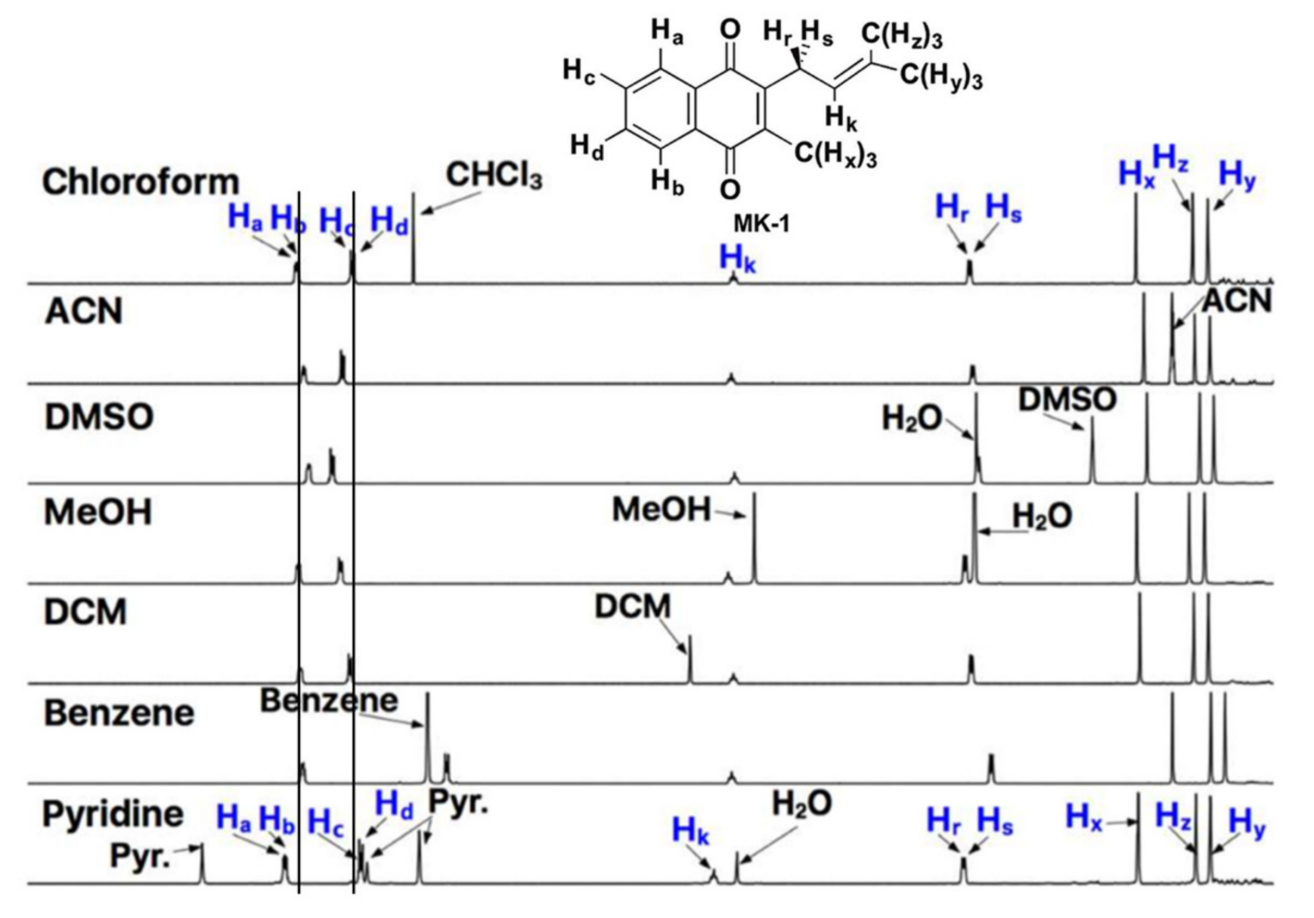
1D NMR spectra of MK-1 in polar and nonpolar solvents. Lines through the aromatic protons, Ha/Hb and Hc/Hd, are provided to highlight the changes in chemical shift. Adapted with permission from *Biochemistry* 2019, 58, 1596–1615. Copyright 2022 American Chemical Society.

Recently, truncated lipoquinones have been used to overcome solubility issues. These derivatives contain the same headgroups as the long-chain counterparts but with shorter sidechains (1–4 isoprene units). For example, MK-2 and UQ-2 have been used as truncated representatives of MK-9 and UQ-10, respectively. Although these short-chain derivatives are still hydrophobic, they are more soluble in organic and aqueous solutions making them attractive alternatives for analysis in aqueous assays. The truncated lipoquinone derivatives are small enough to fit within a typical phospholipid bilayer. One of the first examples of using truncated lipoquinones occurred when Kishi and coworkers measured the reduction potentials of truncated lipoquinone and lipoquinol derivatives in dimethylformamide and calculated their potentials in water [70]. In our recent study, we synthesized MK-1 and MK-1(H_2_) for conformational analysis using 1D and 2D NMR techniques (See Section 2.4 for additional details) [71]. Therein we observed the chemical shifts of the aromatic protons shifted slightly between polar and non-polar solvents. As shown in Figure 4, the aromatic protons Ha/Hb and Hc/Hd shift closer together in polar solvents and spread further apart in nonpolar solvents. Similar studies were reported using MK-2 [60] and UQ-2 [41]. This was supported further through 2D Nuclear Overhauser Effect (NOESY) and Rotating-frame Nuclear Overhauser Effect (ROESY) NMR studies (See Section 2.4.1 and Section 2.4.3) Through the use the truncated derivatives, we were able to investigate the conformations of the lipoquinones not only in organic solvents but also in a model membrane system. Similar studies would not have been possible if the long chain derivatives were used for the NMR study; both because of the complications relating to the assignment of protons on the long sidechain and the fact that the model membrane system has a monolayer and the MKs with longer sidechains would span and then extend past the monolayer of the reverse micellar model system.

Since lipoquinones are sensitive to their environment regarding their structure and their properties, their hydrophobicities suggest that at sufficient concentration these compounds may aggregate and form micelles in aqueous solutions. In our above mentioned studies regarding the conformation of truncated menaquinones (MK-1 and MK-2) and ubiquinone (UQ-2) in several organic solvents, we have not seen evidence for aggregation at the lower concentrations we have been examining these systems [60,71]. However, formation of micelles would be anticipated in aqueous solutions. When these molecules are in polar and non-polar organic solvents, we observed that both UQ and MKs fold over when the side chain is long enough. Since the interactions between the headgroup and one double bond is less than what is anticipated between two molecules of MK-derivatives (the most hydrophobic lipoquinone in this review), we would expect aggregation to take place when the concentration of the MK-derivative is sufficient in a solvent when the solubility is low, even though neither we nor others have direct evidence for such interactions at this time.

### 2.3. Electrochemical Properties

**Scheme 1:**
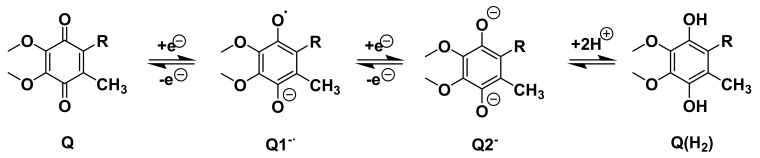
Illustration using a UQ-derivative as an example of a one electron reduction of the quinone (**Q**) to the semiquinone radical anion (**Q1^−^**) and the second one electron reduction to the dianion (**Q2^−^**). Abstraction of two protons results in **Q(H_2_)**.

**Scheme 2:**
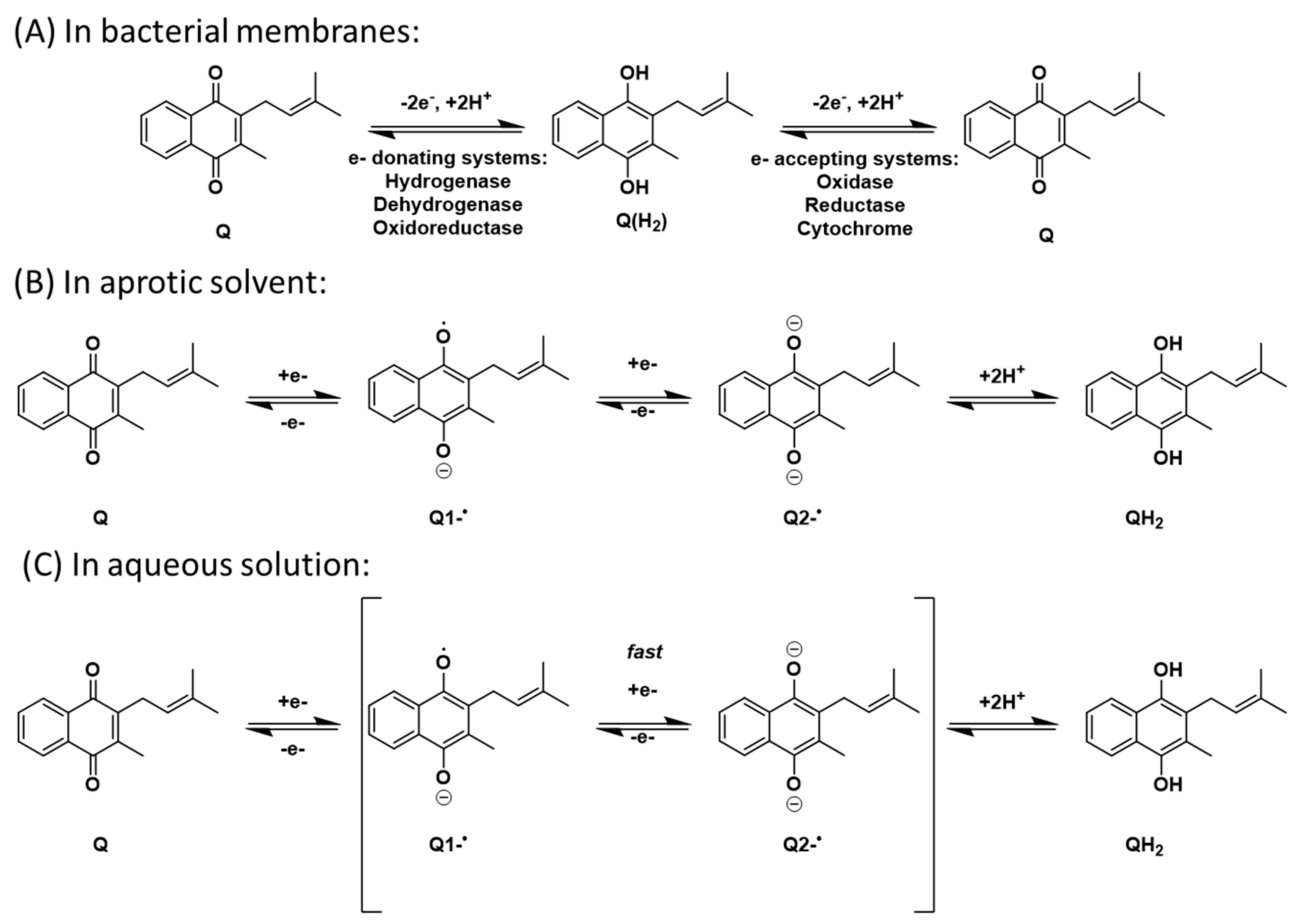
Electron transport mechanisms envisioned for (**A**) bacterial membranes, (**B**) aprotic solvents, and (**C**) aqueous solution.

The quinone headgroup gives lipoquinones their redox activity as electron donors and acceptors in membranes as shown with a UQ-derivative in Scheme 1 and an MK-derivative in Scheme 2 [72,73,74,75,76,77,78,79]. Using “Q” as a general identifier for the redox state of the headgroup, the 1,4-quinone (**Q**) is reduced to the 1,4-quinol (**Q(H_2_)**).The first addition of an electron forms the radical anion, referred to as a semiquinone radical anion (**Q1^−^**). The second addition of an electron generates the dianion catecholate (**Q2^−^**) that ultimately abstracts two protons to form the product, 1,4-quinol (**Q(H_2_)**) [72,73,74,75,76,77,78,79]. The reaction is reversible to allow lipoquinones to traverse between different protein complexes to serve their function as electron transfer agents.

In organic solvents, the number of intermediates (**Q1^−∙^**and/or **Q2^−^**) is dependent upon the proticity of the solvent [72,73,74,75,76,77,78]. For example, both intermediates are observed in aprotic solvents where there are no acidic protons available. The reduction of the quinone in anhydrous aprotic media such as acetonitrile (MeCN), dimethyl sulfoxide (DMSO), or pyridine hence follows a different path than in aqueous solution where protons are available. In protic solvents, including water, only one intermediate is observed. This is due to the radical anion and dianions rapidly abstracting the available protons and cause the two intermediates to be observed as one (Scheme 2A,C). This difference is clearly observed in electrochemical studies by the location of the potentials in the cyclic voltammograms (CV). In biological systems, this reaction occurs in the hydrophobic membrane bilayer. Under these conditions the two different intermediates have finite lifetimes and can be observed; therefore, the bilayer behaves as an aprotic environment (Scheme 2B). Quinone redox chemistry is generally investigated in aprotic solvents, where any differences in redox potentials can be measured reproducibly. We note that these aprotic organic solvents better reflect the hydrophobic regions of the lipid bilayer where lipoquinones are more likely to reside and allow for comparison in hydrophobic environments.

The half wave potentials can be calculated using Equation (1) where E_pc_ and E_pa_ are the cathodic and anodic peak potentials, respectively. The i_pc_ and i_pa_ were measured from the CVs to determine if the reactions are reversible and all ratios equal 1. The number of electrons in each process can be determined using Equations (2) and (3), where ΔE_p_ is the difference between cathodic and anodic peak potentials, which differ by 0.058 V for one electron in non-aqueous solvents.

(1) $$E_{\frac{1}{2}}=\frac{E_{\mathrm{pc}}+E_{\mathrm{pa}}}{2}$$

(2) $$\Delta E_{p}=E_{\mathrm{pc}}-E_{\mathrm{pa}}$$

(3) $$n=x*\frac{0.059\;V}{\Delta E_{p}}$$

**Figure 5:**
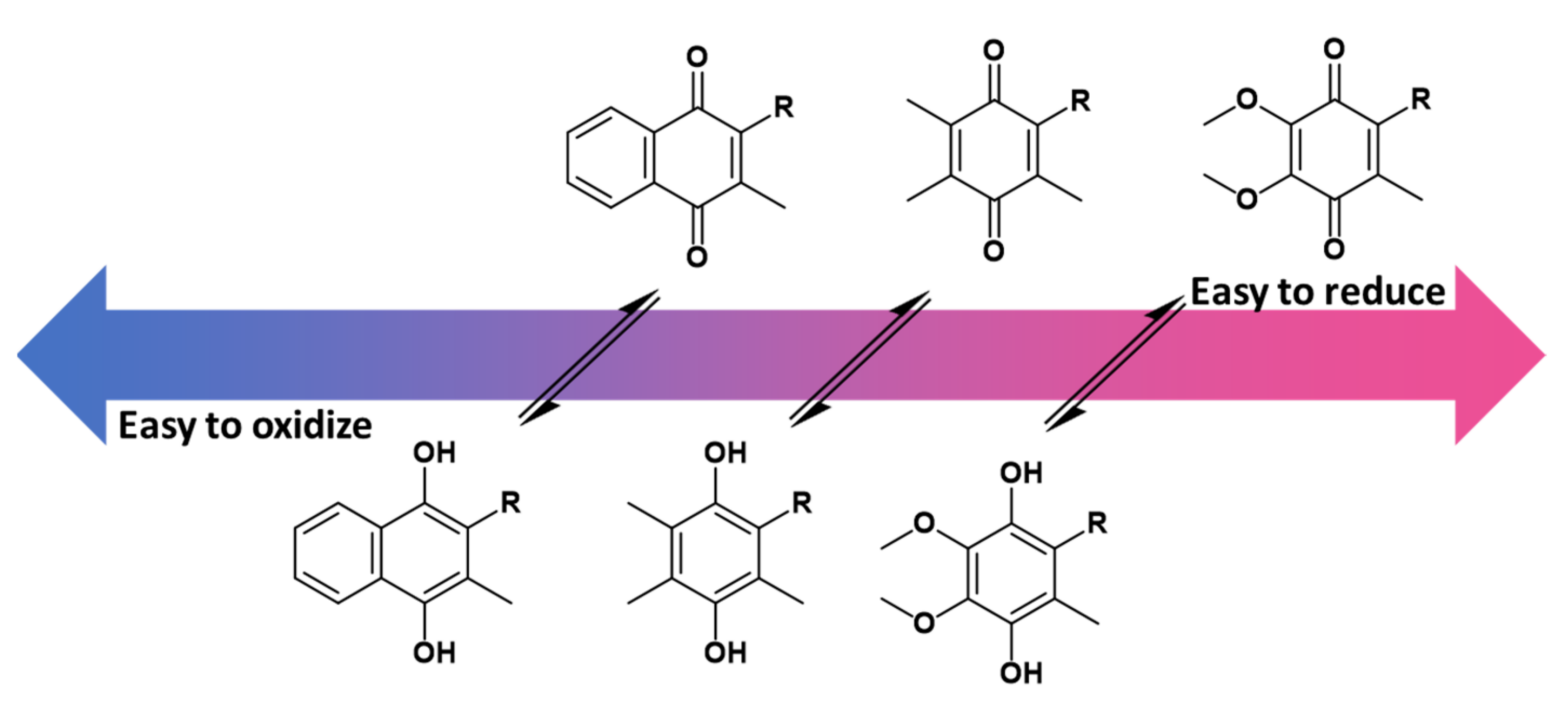
The redox potential of ubiquinone, plastoquinone, and menaquinone are shown relative to one another based on known reduction potential values [80]. Overall, menaquinones are the most difficult to reduce to the quinol form. Therefore, the menaquinol form is the easiest to oxidize back to the quinone. Ubiquinone is the easiest to reduce, and therefore ubiquinol is the most difficult to oxidize. Details of the extrapolations from the experimental data to the redox potential relevant to biological conditions are described elsewhere in detail. The conversion between quinone to quinol is represented with equilibrium arrows, but it is important to note that equilibrium is condition dependent.

Although all three lipoquinones are capable of this redox activity, some undergo reduction and oxidation easier than others because of the nature of the headgroup and sidechain structure. For example, menaquinones are the most difficult to reduce, meaning the corresponding quinols are more easily oxidized (Figure 5). On the other hand, ubiquinones are easiest to reduce, and therefore ubiquinol is more difficult to oxidize. As mentioned in the introduction, the Great Oxidation Event brought on by the increased levels of atmospheric oxygen gave rise to ubiquinones that could withstand the oxidizing environment [14]. According to the reduction potentials of plastoquinones, they would be found in between ubiquinone and menaquinone activity, which is schematically illustrated in Figure 5.

The chemical environment affects the redox reaction, and the hydrophobicity of lipoquinones affect their solubility. Therefore, the chemical environment could impact the number of intermediates observed in the redox reaction. Specifically, whether proton donors are available will determine if the intermediate semiquinone radical anion and dianion are sufficiently long lived and can be observed. In other words, the reaction is observed as one step with no observable intermediate, or as two steps with an observable semiquinone radical anion, respectively The details of how the experimental data is obtained when the reaction is taking place is therefore paramount. Oftentimes values found in tables for reduction potentials in aqueous media have been extrapolated from measurements carried out in organic solvents because the compounds are not soluble in water. It is important to recognize that electrochemical studies are usually measured in the presence of sufficient ions (electrolytes) to measure electron transfer to and from organic molecules. Therefore, the reaction conditions are probably not as close to those actually observed in the biological system where the environment in the membrane is likely to be more hydrophobic. These studies probing the redox reaction are therefore generally done in the absence of water but at high enough substrate concentrations and electrolytes that the redox potentials can be measured. Unfortunately, the experimental limitations are often not discussed and appreciated. If limited details of the measurements nor the extrapolations are mentioned, then comparison of the relevant redox potentials is more circumspect, and, in worst case scenarios, lead to an erroneous conclusion.

While the function of menaquinone derivatives is well established, the role of regiospecific partial saturation in the isoprenyl sidechain on menaquinone’s redox potential remains unclear. The effects of structural differences on the redox potentials and diffusion coefficients of various MK-derivatives in three aprotic solvents (MeCN, DMSO, and pyridine) has been reported in a Ph.D. dissertation by Cheryle Beuning at Colorado State University [79]. This Ph.D. dissertation investigates the partially saturated MK-2(II-H2) and MK-3(II-H2) derivatives, and these were found to be easier to reduce (more positive potentials), than the fully unsaturated and the fully saturated MK-derivatives, the latter being among the hardest MK-derivatives to reduce (more negative potentials). Most unsaturated MK-derivatives were in the mid-range of potentials measured. These results are in line with the interpretation that the composition of the sidechain plays a role in the reduction potential of the compound. In addition, there are examples of stable semiquinone species having biological activity with receptor sites in QH2-reductases; however, the most common reduced form in biological systems is the quinol [81,82,83]. Undoubtedly, these patterns provide insight into these interesting molecules.

### 2.4. Conformation of Lipoquinones

**Figure 6:**
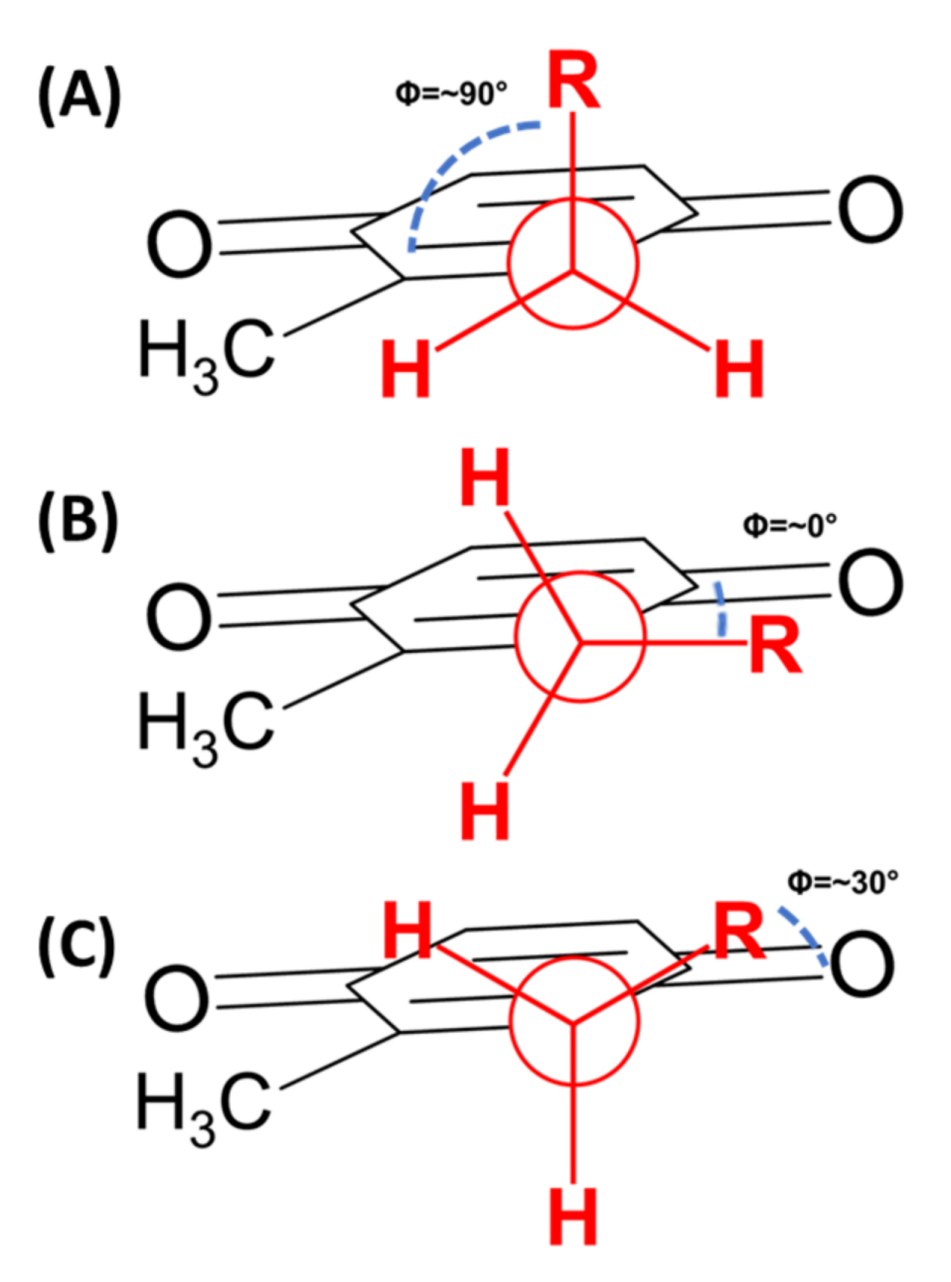
Newman projections of folded and flat conformations. (**A**) Folded conformation with a dihedral angle of approximately 90°. (**B**) Flat conformation with a dihedral angle of approximately 0°. (**C**) Gauche conformation with a representative dihedral angle of ~30°.

As mentioned above, polyisoprenoid molecules have been known to adopt specific conformations in solution. In a study by Murgolo and coworkers, they determined dolichol-19, a polyisoprenyl alcohol with 19 isoprene units, adopts a central coiled conformation flanked by two arms [84]. In lipoquinones, the polyisoprenoid sidechain allows for multiple degrees of freedom to adopt a myriad of conformations. The sidechain’s ability to rotate suggests the sidechain could associate with itself and/or the headgroup of the MK-derivative. To discuss the conformations, we will begin by describing the relationship between the headgroup and the sidechain using approximate dihedral angles about the bond connecting the headgroup to the sidechain (Figure 6). A folded conformation refers to a dihedral angle of ~90° where the sidechain is orthogonal to the headgroup (Figure 6A). A flat conformation refers to a dihedral angle of ~0° where the sidechain is in plane with the headgroup (Figure 6B). A gauche conformation will refer to any dihedral angles in between, shown with a dihedral angle of 60° in Figure 6C.

**Figure 7:**
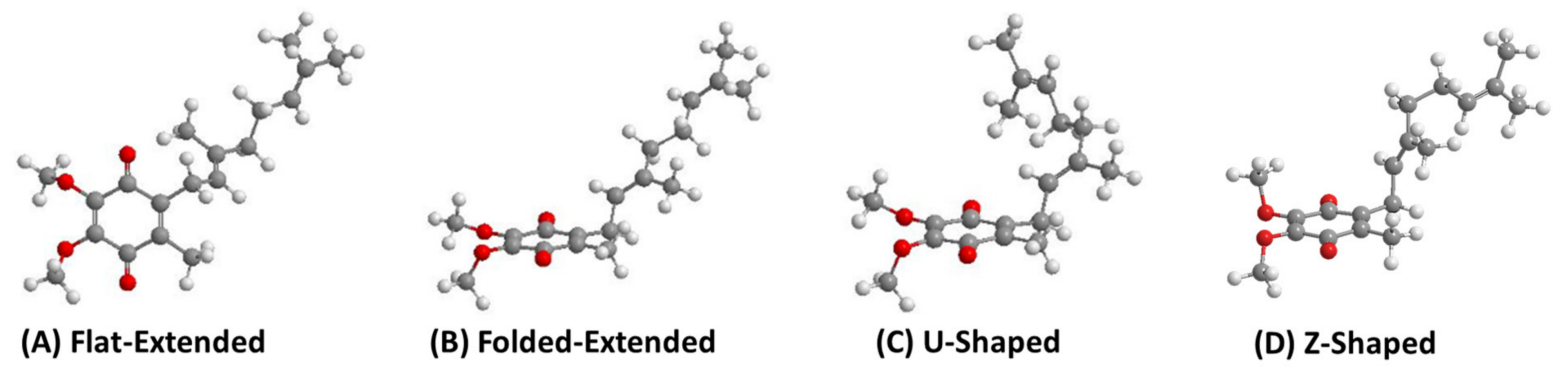
Four different possible conformations, using UQ-2 as an example, describing the relationship between the headgroup and the end of sidechain observed in computational studies. (**A**) Flat-extended conformation, (**B**) Folded-Extended, (**C**) U-Shaped. These images were reported in our recent paper regarding the conformations of UQ-2 [41] and licensed under CC BY 4.0. Here we also include an additional conformation (**D**) Z-shaped, generated using calculations in Chem3D.

The isoprene units along the sidechain have many degrees of freedom to allow for multiple points of rotation that could lead to countless conformations. For simplicity, we will focus on the overall direction the end of the sidechain points relative to the headgroup (Figure 7). When the sidechain extends straight from the headgroup to sidechain bond, this will be referred to as “flat-extended” or as “folded-extended”, as shown in Figure 7A,B. When the end of the sidechain is positioned over the headgroup and is referred to as a U-shaped conformation (Figure 7C). When the sidechain is pointed away from the headgroup, it will be referred to as “Z-shaped” (Figure 7D). Throughout the literature, ubiquinones, plastoquinones, and menaquinones have been shown to adopt some variation of a folded conformation in computational studies [36,52,85,86,87,88], but very few experimental studies have been able to support these findings until recently [41,60,71,89].

#### 2.4.1. Ubiquinones

Several computational studies have investigated the conformation of the headgroup relative to the sidechain by rotating about the bond attaching the sidechain to the headgroup and calculating the energy. Nilsson and coworkers determined the dihedral angle of UQ-et, an ethyl substituted UQ-derivative, to be ~90°at a global energy minimum [87]. Ceccarelli and coworkers found similar results in their study using UQ-1, which showed two local minima: ~90°and ~270°, both orthogonal relationships [86]. Both studies support a folded conformation of the UQ-derivatives regardless of the type of sidechain. In contrast, Galassi and Arantes found the dihedral angle of UQ-1 to be ~180°, supporting a flat-extended conformation. Since all three studies were computational, it is difficult for the non-expert to determine if the differences between the studies were due to variations in parameters used or if the energy barriers are actually that low to rotate between flat and folded conformations [36]. In addition, other studies only investigating the location of lipoquinones in membrane environments reported figures that showed folded conformations, and whether or not this reflected in detailed conformational data is unknown [38,63,90,91,92]. Experimental studies are then necessary to validate the computational findings.

**Figure 8:**
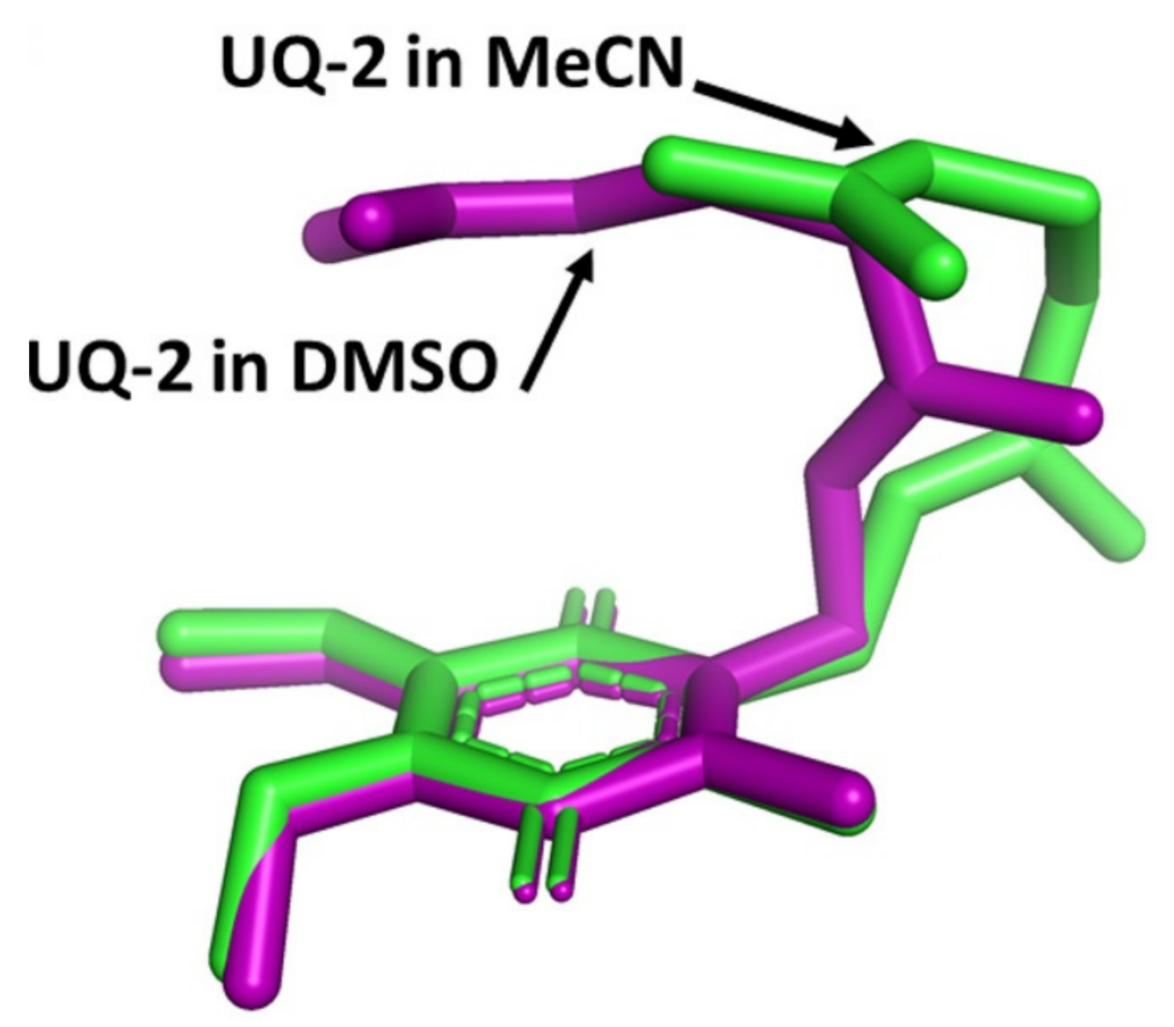
Superimposed 3D conformations of UQ-2 based on the 2D NMR data in d_3_-acetonitrile (MeCN, green) and d6-DMSO (purple). This figure was originally published in Ref. [41] and is licensed under CC-BY 4.0. The original figure included two images however only the depicted image is shown here.

In our recent study, we determined the conformation of UQ-2 in organic solvents using 2D ^1^H-^1^H NOESY and ROESY NMR spectroscopy [41]. For example, the presence of a cross peak between the headgroup methyl protons and the protons along the sidechain suggest they must be within ~5 Å to cause magnetization transfer between these two protons. Investigating conformations in polar and nonpolar organic solvents, such as DMSO, acetonitrile, and pyridine, we observed cross peaks that suggest UQ-2 adopted more of a U-shaped conformation in polar solvents. A clear indication of a U-shape verses the other possible conformations is a cross peak between the methyl group of the headgroup and the geminal dimethyl groups at the end of the sidechain, which will be referred to as the terminal methyl groups. To better visualize the differences between UQ-2 in acetonitrile and DMSO, we calculated the distances between the atoms using the 2D NOESY and ROESY data (for additional details see [41]) to build the conformations, and the resulting conformations were superimposed and are shown in green and purple, respectively, in Figure 8. With the headgroups aligned, there is a slight change in dihedral angle along the bond connecting the headgroup to the sidechain. As the sidechain extends, this change in dihedral angle has considerable influence on the position of the terminal methyl groups. In DMSO, the terminal methyl groups are located above the headgroup, almost parallel, leading to a U-shaped conformation. In acetonitrile, the terminal methyl groups are angled away from the headgroup leading to a more gauche conformation of the sidechain. In nonpolar solvents, such as benzene and pyridine, cross peaks between the headgroup methyl and the vinyl protons along the sidechain suggest UQ-2 adopts a folded conformation as well, but there were no cross peaks between the headgroup methyl and the terminal methyl groups. This indicates UQ-2 adopts a folded conformation in nonpolar solvents, but the sidechain is extended or positioned as more of an open U-shaped conformation.

In our recent study, we also determined the conformation of UQ-2 in a model membrane system bis(2-ethylhexyl)sulfosuccinate sodium reverse micelles (abbreviated AOT RMs) using 2D ^1^H-^1^H NOESY and ROESY NMR spectroscopy [41]. When UQ-2 was added to AOT RMs, cross peaks between the headgroup methyl group and the terminal methyl groups were observed, suggesting a U-shaped conformation. It would make sense that the UQ-2, when incorporated in a membrane, would adopt a more compact structure akin to the structure observed in d_6_-DMSO. The full details on this study can be found in our publication [41].

#### 2.4.2. Plastoquinones

Computational studies have shown plastoquinones adopting folded conformations in membrane [52,89] and solvent environments [85,88]. A study by de Jong and coworkers determined the dihedral angle of plastoquinones were 100° and 250° in a membrane environment, supporting a folded conformation [52]. Himo and coworkers investigated the conformation of PQ-et, UQ-et, and MK-et to understand how the C2 methyl group on the headgroup impacts rotational freedom of the sidechain in solvent environments [85]. In the presence of the methyl group, the dihedral angle of menaquinone and ubiquinones was found to be ~100° within a clear energy minimum along the indicated bond. This is consistent with a folded conformation. However, in the case of PQ-et, the local energy minimum was preceded by a low energy plateau from 0–100°. Nilsson and coworkers came to a similar conclusion [88]. Although the energy minimum at 100° suggests the folded conformation is preferred, the plateau implies the sidechain of PQ-et can rotate freely between flat and folded conformations without energy penalties. Considering the structure of each headgroup, plastoquinones do not contain the C2 methyl group; therefore, the C2 methyl group must behave as a rotational barrier to influence their conformations.

In addition to the conformation of the headgroup relative to the sidechain, de Jong and coworkers also investigated the preferred conformations of the sidechains within a simulated lipid bilayer using two different computational approaches: United Atom (UA) and Coarse Grain (CG) [52]. Within both datasets, the sidechains were observed in three distinct conformations that they called U, L, and I conformations. In the U conformation, the sidechain extends into the midplane and then curves back towards the headgroup, intercalating the end of the sidechain into the acyl tails. The L conformation placed the end of the isoprenoid sidechain within the bilayer midplane, forming an approximate right angle. The I conformation extends the sidechain through the midplane into the opposite leaflet. In their simulations, they observed different populations of each conformation for the same lipoquinones. For example, the sidechain of PQ-9 was found to adopt L:I:U ratios of 53:16:13 and 46:18:13 for UA and CG studies, respectively. Overall, both computational approaches showed the L conformation was the most popular conformation, followed by I, and then U. The preference for the L conformation suggests the sidechain is embedded in the midplane to minimize interactions with polar environments.

#### 2.4.3. Menaquinone

Of the three lipoquinones we examine in this review, the properties of menaquinones have received the least attention in the literature. In our recent publication, we reported an analogous study to the UQ-2 study described in Section 2.4.1 with truncated, MK-derivatives, MK-2, MK-1, and MK-1(H_2_), in organic solvents using 1D and 2D NMR spectroscopy [60,71]. Like UQ-2, MK-2 adopts a folded, U-shaped conformation in polar solvents, such as DMSO and acetonitrile. MK-2 was found to adopt a folded-extended or a more open U-shaped conformation in nonpolar solvents, such as benzene or pyridine. The details regarding the conformational determination of MK-2 in organic solvents can be found in our publication [93].

The conformation of MK-2 in AOT RMs was similarly determined by the cross peaks between the headgroup and the terminal methyl protons suggest that the end of the sidechain is positioned over the headgroup, overall suggesting MK-2 adopts a folded, U-shaped conformation in AOT RMs. The conformations of MK-1 and MK-1(H_2_) were also investigated using analogous NMR studies [71]. Both derivatives were found to adopt folded-extended conformations in organic solvents and AOT RMs. This designation was attributed to the shorter sidechain that is not long enough to extend over the headgroup to achieve a U-shaped conformation.

In a recent study by Sitkowski and coworkers, MK-7 derivatives found in dietary supplements were characterized using multidimensional NMR analysis [94]. The researchers also investigated the conformations of each isomer using DFT modeling studies. The resulting conformations show each MK-7 isomer is folded, showing an approximate 90° dihedral angle about the bond connecting the headgroup to the sidechain. The position of the sidechain seems to be dependent upon the geometry of the double bonds. By the definitions established at the beginning of this section, the sidechain of (E6,ω)-MK-7 adopts a folded-extended conformation. Interestingly, the sidechain of the (Z6,ω)-MK-7 isomer adopts a helical conformation that is extended away from the quinone headgroup. The authors note the similarities between the helix observed in the sidechain and the observed coiled conformations of dolichol [84]. Finally, the (E, Z3,ω)-MK-7 isomer adopts a less defined conformation along the sidechain. Overall, the derivative is folded, and the sidechain extends away from the headgroup for 2–3 isoprene units, adopting part of a helix at the Z3-isoprene unit. From there, the sidechain appears to extend further after the turn of the helix. This study suggests the conformation of the sidechain is dependent upon the geometries of the double bonds within the sidechain.

### 2.5. Comparison of Lipoquinone Conformations

The C2 methyl group adjacent to the sidechain impacts the conformation of lipoquinones. In a recent study by Eddine and coworkers, combined the results of DFT modeling and EPR spectroscopy, specifically ^1,2^H hyperfine coupling constants, were used to evaluate the steric effects of the headgroup methyl group of long chain UQ-, MK-, and PQ-derivatives [89]. Their results corroborate the findings of Himo and Nilsson and support the hypothesis that the headgroup methyl group behaves as a rotational barrier to cause lipoquinones to favor a folded conformation. Each energy diagram of UQ- and MK-derivatives have a distinct energy minimum around 90°. However, as described in Section 2.4.2, PQ-derivatives, showed an energy minimum around 100° with a low energy plateau leading up to it [85]. These differences are consistent with the notion that implies the headgroup structures were selected to afford specific conformations and that those differences could have implications in their functions.

**Figure 9:**
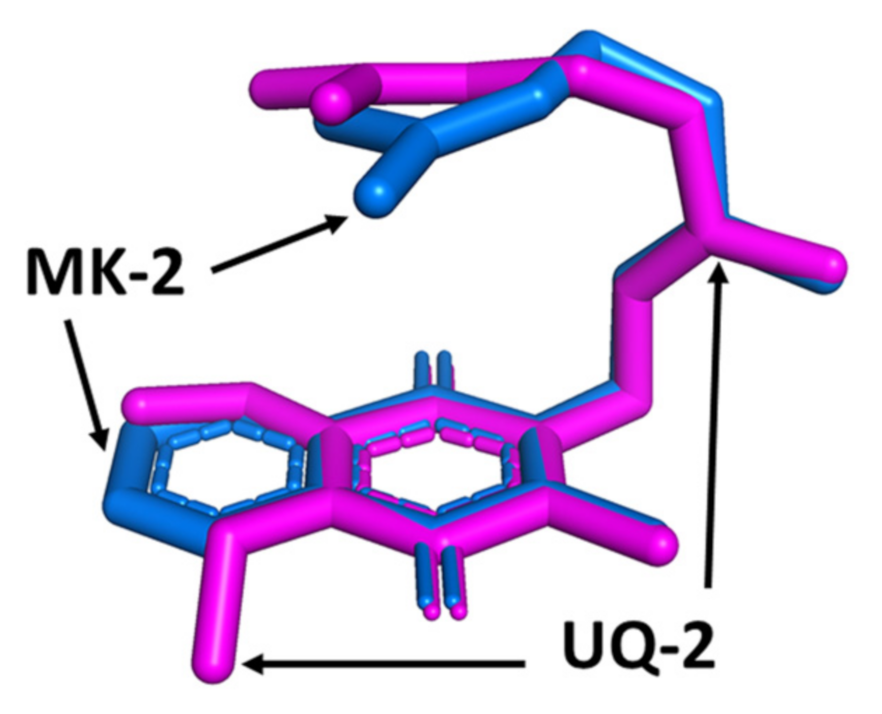
Superimposed 3D conformations of UQ-2 (pink) and MK-2 (blue) based on the 2D NMR data in DMSO. This figure was originally published in Ref. [41] and is licensed under CC-BY 4.0. The original figure was modified to include only the depicted image.

These different results demonstrate that the conformations of lipoquinones are dependent on the chemical environment. As described in the previous sections, MK-2 and UQ-2 are sensitive to their solvent environment, adopting increasingly folded conformations in polar solvents and more open, folded conformations in nonpolar solvents [41,60]. To compare the conformations, we superimposed the conformations of UQ-2 and MK-2 in DMSO to clearly show how the headgroup affects the conformation in the same solvent (shown in pink (UQ-2) and blue (MK-2) in Figure 9). With the headgroups aligned, the sidechains nearly overlap completely until just after the first isoprene unit. The adoption of these folded conformations is likely due to the hydrophobic effect as well as noncovalent interactions such as π- π stacking, where the π bonds in the headgroup are interacting with the π bonds of the sidechain. These results suggest ubiquinones and menaquinones will adopt similar conformations when the chemical environments are similar. Considering a membrane, the hydrophobic effect could greatly influence the conformation of UQ-2 and MK-2 in the more polar region of the membrane. If the lipoquinones were located closer to the bilayer midplane, the nonpolar environment may influence the conformations to adopt more open folded or even flat conformations in line with the observed trend in the model studies.

### 2.6. Conformations of Lipoquinones in Protein Active Sites

**Figure 10:**
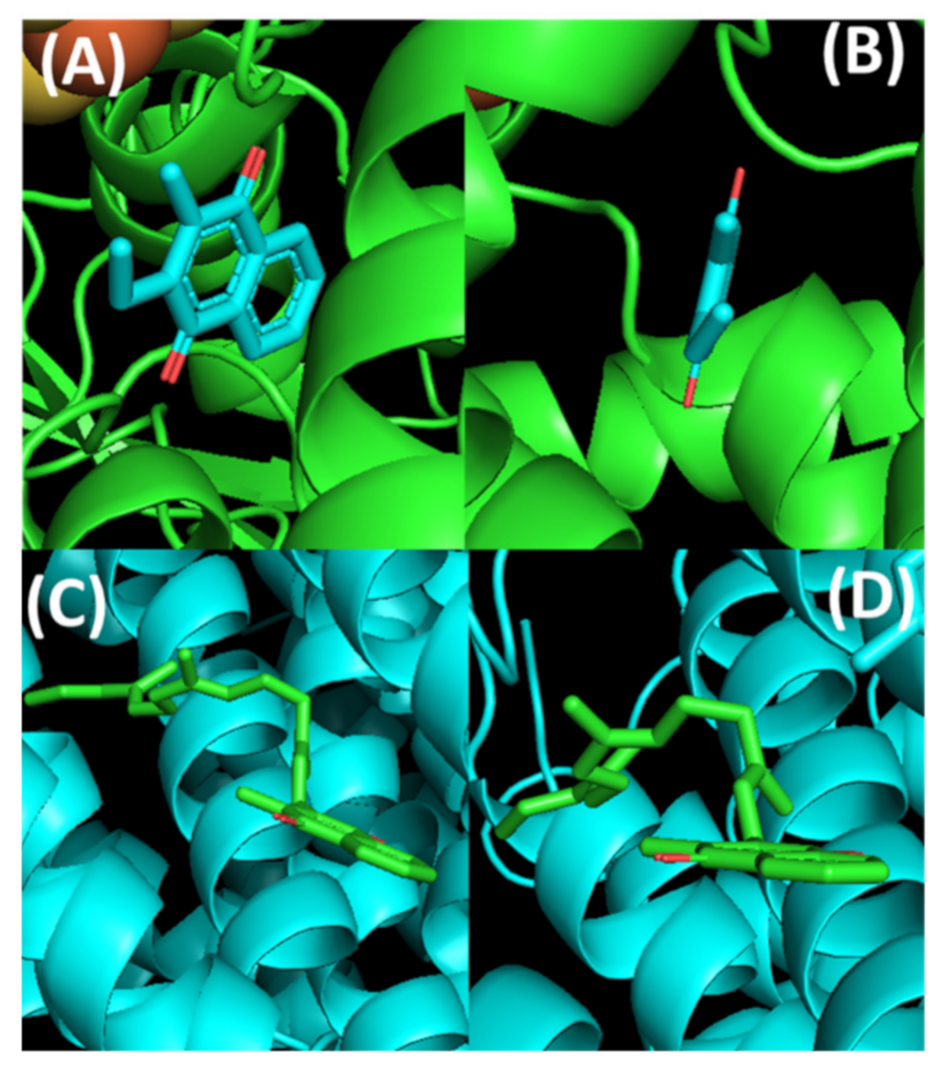
Conformations of MK-7 within the active sites of two different enzymes: Top: *Thermus thermophilis HB27* (PDB ID: 2VPW), and Bottom: *Bacillus subtilis* (PDB ID: 6KOB). (**A**) MK-7 (shown in cyan) full view. (**B**) MK-7 side view showing the plausible flat conformational relationship between the headgroup and the sidechain. (**C**) MK-7 (shown in green) full view highlighting the Z conformation. (**D**) MK-7 side view to highlight the folded conformation along the bond connecting the headgroup to the sidechain. The images were created in PyMOL using the respective PDB codes.

Although the conformations of lipoquinones in organic solvents and membrane environments are very different than lipoquinone conformations associated with proteins and protein active sites, the function of lipoquinones involve proteins and their associations with lipids. Even in the cases of protein-associated lipoquinones, the conformations about the bond connecting the headgroup to the sidechain appears to be specific to the biological system at hand. For plastoquinone, the conformation of the headgroup relative to the sidechain of PQ_A_ within Q_A_ binding site of photosystem II depends on the species of bacteria. The dihedral angle was found to be −10° in *Thermosynechococcus elongatus* [95], 88–89° in *Pisum sativum* [96] and ranging from 32–70° in *Thermosynechococcus vulcanus* [97]. This variation suggests the conformations are influenced by the active site and consequently its specific function, perhaps tailoring the reduction potential of the plastoquinone to the target protein and individual species. This complementarity has also been observed in the lipoquinones cofactor binding sites. For example, MK-7 is an electron carrier in many species of bacteria. Within the reported crystal structure of polysulfide reductase active site of *Thermus thermophilis HB27* (Protein Data Bank (PDB) ID: 2VPW), the MK-7 ligand was simplified to show only part of the first isoprene unit deposited in the PBD crystal structure, which is shown in Figure 10A,B [98]. From this crystal structure, MK-7 appears to adopt a flat conformation, but it is impossible to determine the position of the sidechain from the simplified representation. Within the active site of the menaquinol oxidase of *Bacillus subtilis* (PDB ID: 6KOB)*,* MK-7 was found to adopt a folded conformation with the sidechain pointed away from the headgroup, resulting in a Z shape (Figure 10C,D) [99]. Clearly, the active site is stabilizing the sidechain to adopt a Z-shaped conformation, which is observed less frequently in the membrane. Together these two examples of protein-lipoquinone complexes show that for protein-associated lipoquinones there is a greater variety in lipoquinone conformations than when the lipoquinone is in the membrane. This also sheds light on how much information may be missing by omitting the full lengths of the sidechain when interpreting active site associations. Furthermore, it is possible that lipoquinones possess a baseline reduction potential which can be manipulated by their surroundings to tailor the reduction potentials required to transfer the electrons from the respective proteins.

In addition to the conformation of the sidechain, ubiquinones contain two additional functional groups, methyl ethers, which can be manipulated by its surroundings. For example, ubiquinones have been shown to adopt slightly different orientations between quinone binding sites, Q_A_ and Q_B_, along the electron transport chain of eukaryotes [100]. The differences in dihedral angle have been calculated for each binding site throughout the literature. Nonella and coworkers found a difference of <30° for the out-of-plane methoxy group between Q_A_ and Q_B_ in *Rhodobacter sphaeroides* [100]. Later, Wraight and coworkers determined the dihedral angles of the Q_B_ C2 and C3 methoxy groups to be −90 ± 9°and 88 ± 20°, respectively, in the same bacterium [101]. Taguchi and coworkers determined the C2 methoxy group of Q_B_ is 20–25° more out of the plane of the headgroup than Q_A_, which places it approximate 50 or 155° relative to the headgroup [102]. These conformational changes were found to affect the reduction potentials within the active site, which further suggests conformational changes finetune the reduction potentials of the lipoquinone to the active site.

## 3. Location of Lipoquinones in Membranes

The different structures of the three lipoquinones seem to provide an opportunity to compare the effects of the headgroup on its position within lipid bilayers. However, since lipoquinones have been investigated with a range of different liposome compositions and analytical methods, a direct comparison is somewhat difficult. Furthermore, many experimental and computational studies have been performed with ubiquinones, followed by plastoquinones, but far fewer studies have been done to determine the location of menaquinones in lipid bilayer.

### 3.1. Ubiquinones

The location of ubiquinones in the biological membrane has been of interest to researchers for many years. It has been studied using a variety of experimental and computational techniques that are summarized in Table 3. Although determining the location has caught the attention of researchers, there has not been a consensus regarding its exact position. As a result, the benzoquinone headgroup has been found to be in all three sections of the lipid bilayer. Unfortunately, experiments using similar methods have been reported to produce conflicting results. To highlight a few examples, two studies reported using chiral shift NMR spectroscopy. Specifically, Kinglsey and Feigenson used a deuterated model membrane system and Dy^3+^ and Tm^3+^ chiral shift reagents to investigate the location of UQ-10 [33]. They observed the chemical shifts of the methoxy groups were influenced by the presence of the chiral shift reagent. This led to the conclusion that the headgroup must be close enough to the interface to interact with the chiral shift reagent in the aqueous environment. On the other hand, Michaelis and Moore used Pr^3+^ & Eu^3+^ chiral shift reagents and found the chemical shifts were not affected by the chiral shift reagents. This led to the conclusion that the headgroup must be far away from the interface, at least past the C2 carbon of the acyl chains within the lipid tails [42]. Although these studies used the same analytical method, it is important to note they used different model membrane systems: Kingsley and Feigenson used dimyristoylphosphatidylcholine-*d*72 (DMPC-*d*72) and Michaelis and Moore used egg phosphatidylcholine (EPC). Furthermore, the two studies used different chiral shift reagents. It is possible the contradicting data could be a result of the model membranes or chiral shift reagents used.

Overall, many of the discrepancies between the studies can be attributed to the methods used and the varying detections limits between methods. Primarily, it is critical to consider the hydrophobicity of lipoquinones when approaching the formation of lipid vesicles. It is also important to acknowledge the solubility of the analytes used in each study, the complexity of these systems, and the purity of the lipids used in the study. Most of the experimental studies listed in Table 3 formed the lipid vesicles using a similar procedure. The analyte was dissolved in a polar solvent, like chloroform, and the lipids were added to the mixture. The solvent was removed and then rehydrated with D_2_O. It is important to note the variability in the concentration of lipoquinone and the ratio of lipids present in each study. The final membrane structure (lipids + lipoquinones) is a property of the mixture; therefore, the molar fraction of the lipoquinone and the composition of the model membrane are important when interpreting the results. It is also important to note that most of the studies in Table 3 used UQ-derivatives with normal, isoprenoid sidechains.

Two studies, on the other hand, utilized modified sidechains. Chazotte and coworkers attached a fluorescent tag, NBDHA (6-(N-(7-nitrobenz-2-oxa-1,3-diazol-4-yl)amino)hexanoic acid) to the UQ headgroup with a ten carbon chain [44]. Gomez-Murcia and coworkers used idebenone, a UQ-derivative with a ten carbon chain terminating in a hydroxy group [37]. These modified sidechains introduce more opportunities for intramolecular and intermolecular noncovalent interactions. Lastly, three computational studies were reported [36,40,51], and there can be a lot of variety within the parameters used in a computational experiment, such as logP values, as discussed in Section 2.1. As the field moves forward, experimental approaches that can circumvent the predictable issues of solubility are needed to allow for more direct comparisons between systems.

Recently, a Q binding protein, abbreviated Coq10, has been reported in eukaryotes (*Saccharomyces cerevisiae*) [103,104] and in mitochondria in human beings where two orthologs [105] have been reported (Coq10A and Coq10B). The protein is a member of the steroidogenic acute regulatory protein (StAR)-related lipid transfer (START) domain superfamily. The physiological roles of this protein have been documented with deletion mutants that are respiratory defective and sensitive to oxidative stress. Supplementation of Q has been reported to alleviate aging symptoms and mitochondrial deficiency syndrome [106,107]. This protein is found to bind one molecule of Q either in the oxidized and reduced state. It has been proposed to be a Q chaperone and deliver both the oxidized and reduced forms of Q between the respiratory complexes and hence contribute to impacting the location of Q in the membrane and is described here. Several Q derivatives, including affinity labels, were found to bind in a hydrophobic channel of this protein [103,107] leading to the suggestion that Q may be associated with this protein and not free in the membrane. These findings could be considered in contrast to the notion that a “pool” of free Q exist in the membrane and move between the respiratory complexes [36,40,52,53,108,109,110,111]. However, considering that lipoquinones also have antioxidant effects [5] and are small molecules, it seems likely that there are both Coq10-bound and free Q in the membrane.

### 3.2. Plastoquinones

Plastoquinones are located within the thylakoid membrane of chloroplasts. The exterior stroma and interior lumen of the thylakoid membrane are comprised of aqueous media, so the consensus seems to be that the hydrophobic plastoquinones molecules form a pool, freely diffusing throughout the bilayer midplane [111,112,113]. However, some computational studies have shown the plastoquinone pool is not one large pool throughout the midplane but rather a number of small pools throughout. Computational studies have shown higher concentrations near proteins [55,57,109]. On the other hand, recent computational studies have shown plastoquinone located near the polar lipid headgroups. That is, van Eerden and coworkers characterized the interactions of cofactors found in photosystem II in different lipid systems present in thylakoid membranes, such as phosphatidylglycerol, digalactosyldiacylglycerol, monogalactosyldiacylglycerol, and sulfoquinovosyldiacylglycerol [53]. Through their efforts, plastoquinone was found near the glycerol section of the polar lipid headgroups with the sidechain pointing towards the midplane [53]. Additionally, de Jong and coworkers performed a simulation of PQ-9 and other lipoquinones in a DPPC lipid bilayer. Therein they determined the headgroup of PQ-9 was located at or near the lipid headgroup [52]. These computational studies came to the same conclusion; however, there is currently not yet experimental evidence to support their findings.

### 3.3. Menaquinones

Recently, our group used truncated MK-derivative, MK-2, to shed light on the membrane location using AOT reverse micelles as a model membrane system using 2D ^1^H-^1^H NOESY and ROESY NMR spectroscopy [60]. Cross peaks were observed between aromatic headgroup protons of MK-2 and protons near the polar headgroup of AOT. Together these results suggest the menaquinone headgroup is located near the lipid-water interface of the AOT RMs [60]. Protons along the sidechain of MK-2 formed cross peaks with protons along the alkyl chains near the polar headgroup of AOT, which further support the location near the polar headgroup of AOT. In an analogous study, we determined MK-1 and MK-1(H_2_) were also found near the interface of AOT reverse micelles [71]. 

**Figure 11:**
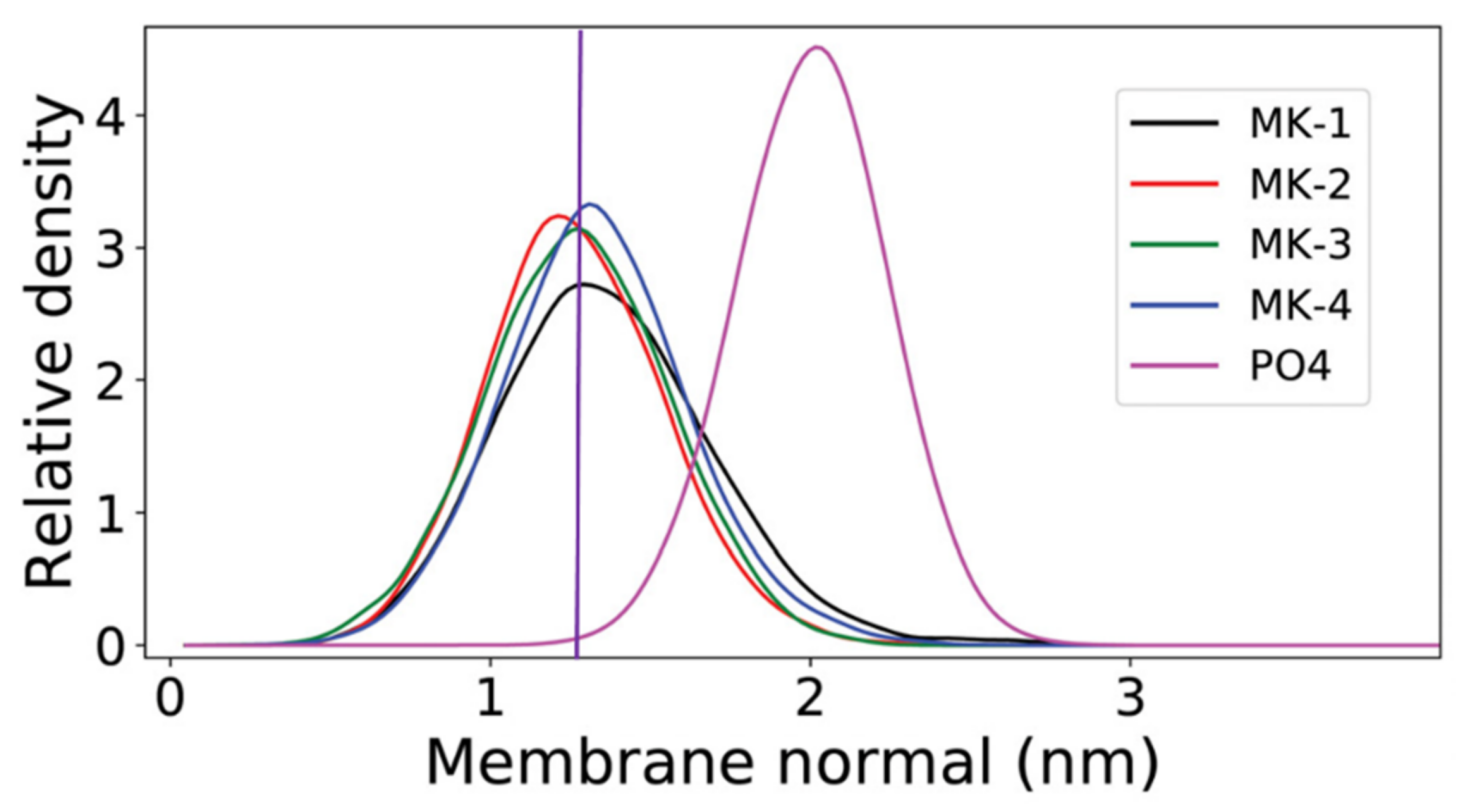
Relative mass density of the menaquinone headgroup along the membrane normal for MK-1 (black), MK-2 (red), MK-3 (green), and MK-4 (blue). The phosphate group of POPC (PO_4_) is shown in magenta. The normal zero corresponds to the center of the bilayer. This figure was originally published in Ref. [1] and is licensed under CC-BY 4.0. The original figure was modified to include the purple, vertical line.

The location of MK-1 through MK-4 was determined using Langmuir monolayer studies and in computational study performed by our collaborators, Arantes and coworkers. Using dipalmitoylphosphatidycholine (DPPC) and dipalmitoylphosphorylethanolamine (DPPE) phospholipids, all the truncated MK-derivatives were found to migrate from the air-water interface into the acyl tails at physiological surface pressure, showing the MK-derivatives do associate, but do not disrupt, the phospholipid packing [1]. This contrasts with the results found for UQ-derivatives in a similar experimental study [114,115,116]. In the accompanying computational study, Arantes and coworkers used palmitoyloleoylphosphatidylcholine (POPC) model membrane to determine the location of MK-1 through MK-4. They found that all four headgroups were localized near the polar lipid headgroups of POPC with only slight variations in position as highlighted in Figure 11 [1].

Together these experimental and computational experiments suggest the lipoquinone headgroup has greater influence over the position of the molecule in the membrane than the length of the sidechain and challenges the “Dog vs. Tail” metaphors that have been used to explain the influence of the headgroup versus the influence of the sidechain that was first described by Joela and coworkers [92]. The prevailing theory that the sidechain has the major influence over the position of the headgroup has been described to behave like a tail wagging the dog, where the tail is stationary and the headgroup moves around. The opposite would be the dog wagging its tail, where the headgroup remains in a constant position, and the tail is mobile. The studies by Arantes shows the headgroup structure is important in determining the location of the headgroup, and the sidechain is only influential in positioning the terminus of the sidechain. Combined, these works support the notion that the dog is wagging the tail and not the reverse; the headgroup does not change location with changes in sidechain length, whereas the sidechain will change location with sidechain length. Sidechain length will only determine the position of the sidechain, not the headgroup.

### 3.4. Comparison of Lipoquinones Headgroup Locations

The prevailing opinion in the literature is that the length of the isoprenoid sidechain has the major influence over the location of the quinone headgroup within a membrane bilayer. Although popular, this conclusion is rooted in computational studies supported by some experimental studies and series of data which leaves open the possibility that the headgroup may also be important for location in the membrane. The influence of headgroup type on location has only recently gained popularity fueled by computational studies. To validate the location of the lipoquinone headgroup in membrane environments, more experimental data is needed to support the computational findings and hence challenge the predominant theory that the length of the sidechain has the most influence.

**Figure 12:**
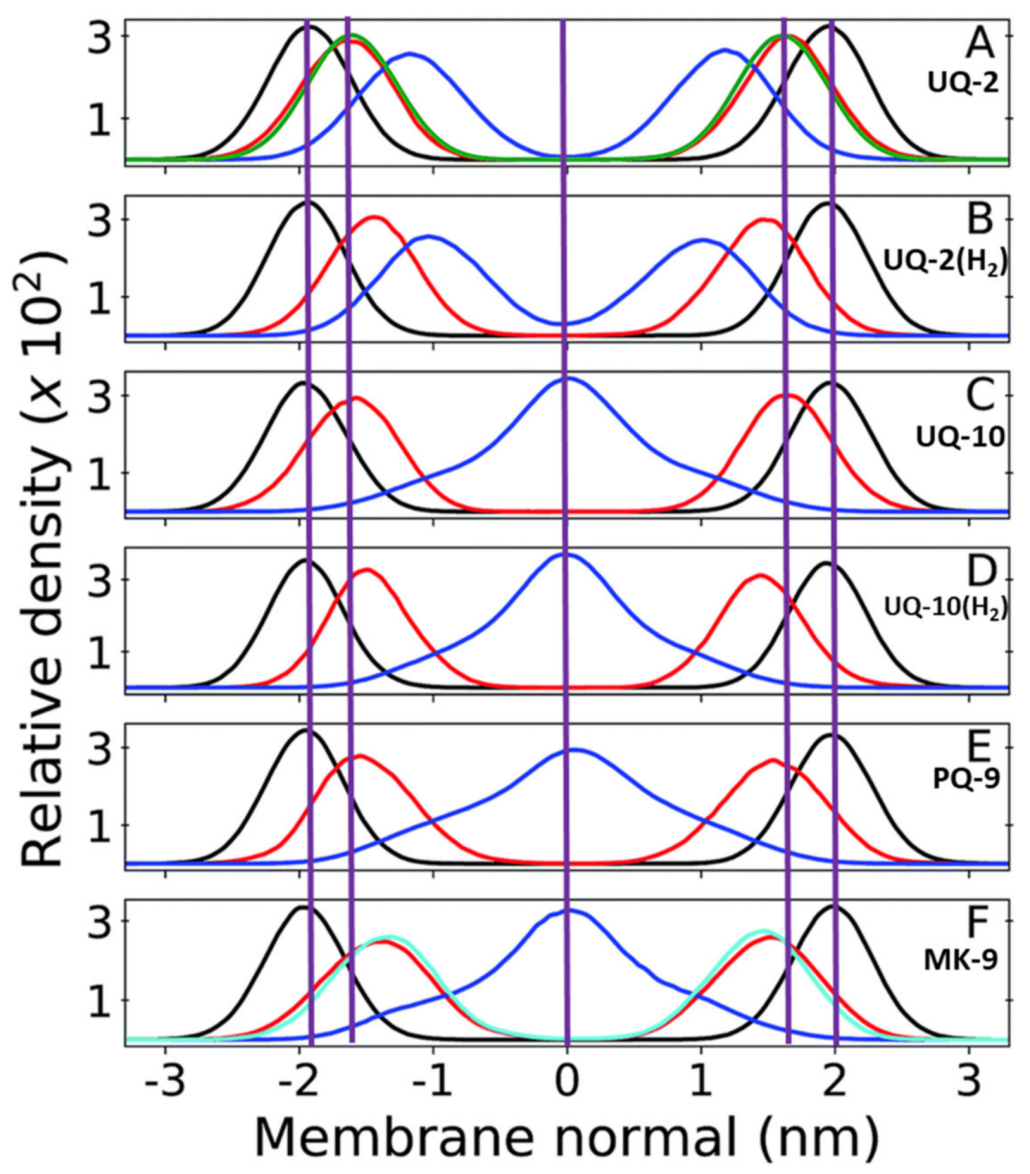
Relative mass density of multi-component bilayers with different lipoquinone derivatives (different lengths, degrees of unsaturation, and headgroups). The phosphate groups of DPLC and DLPE are shown in black, quinone headgroup in red, and sidechain in blue. Vertical purple lines are shown to emphasize the changes in position of the headgroup and the end of the sidechain relative to UQ-2 and the membrane center line. (**A**) UQ-2. Green curve shows the density of the DPLC and DPLE glycerol group; (**B**) ubiquinol-2 (UQH_2_-2); (**C**) UQ-10. (**D**) ubiquinol-10 (UQH_2_-10).; (**E**) PQ-9; and (**F**) MK-9. The cyan line shows the first 6 carbons of the naphthoquinone ring. The red line is the full ring system. The purple zero line corresponds to the center of the bilayer. Adapted with permission from Ref. [40]. Copyright 2022, Royal Society of Chemistry. The figure was modified to include the purple, vertical lines and lipoquinone derivative labels.

In addition to the menaquinone study mentioned in Section 3.3, Arantes and coworkers have investigated the location of other lipoquinones in the membrane using similar computational methods [36,40]. Using lipoquinones of different headgroup type, redox state, and sidechain length, they calculated the distance of the phosphate groups, headgroups and sidechain from the membrane center (Figure 12). When comparing the polar phosphate and quinone/quinol headgroups of the mixed bilayer of dilinoleoylphosphatidylcholine (DLPC) and dilauroylphosphoethanolamine (DPLE), the positions of the quinone and quinol sidechains do change when the length of the chains change (as illustrated by the center purple line in Figure 12). When comparing UQ-2 and UQ-10 (Figure 12A,C) the position of the phosphate groups in the headgroup do not seem to be significantly affected by the increased sidechain length and only slightly when the redox state of the lipoquinone change. The redox state of the lipoquinone headgroup does seem to affect the position of the headgroup slightly. For example, the UQH_2_-2 quinol headgroup is slightly closer to the membrane central plane than UQ-2, but both are still near the phospholipid headgroups (Figure 12A,B). In the case of the headgroups (Figure 12C,D), the position of the UQ-10 headgroup is in nearly the same position as the headgroup in UQ-2, but a change in redox state does seem to affect its position slightly. As discussed in Section 2.1, lipoquinols are more hydrophobic than lipoquinones and could explain why the quinol headgroup is found slightly closer to the midplane of the membrane.

Between UQ-10, PQ-9, and MK-9, the headgroup seems to be slightly closer to the midplane as the polarity of the headgroup decreases, respectively (Figure 12C,E,F). Although this computational study does not include the respective quinols of PQ-9 and MK-9, one could expect them to be slightly closer to the membrane center according to the trend observed with the ubiquinones and the ubiquinols. Together these computational results suggest the nature of the headgroup influences its position in the membrane slightly, but the length of the sidechain does not affect the location of the headgroup or the phosphate group. The location of the sidechain is dramatically influenced by the length of the sidechain and not by the headgroup or the phosphate group. The prevalent theory that the length of the sidechain has the most influence is therefore only valid for the location of the sidechain and not for the locations of the headgroup or phosphate groups.

**Figure 13:**
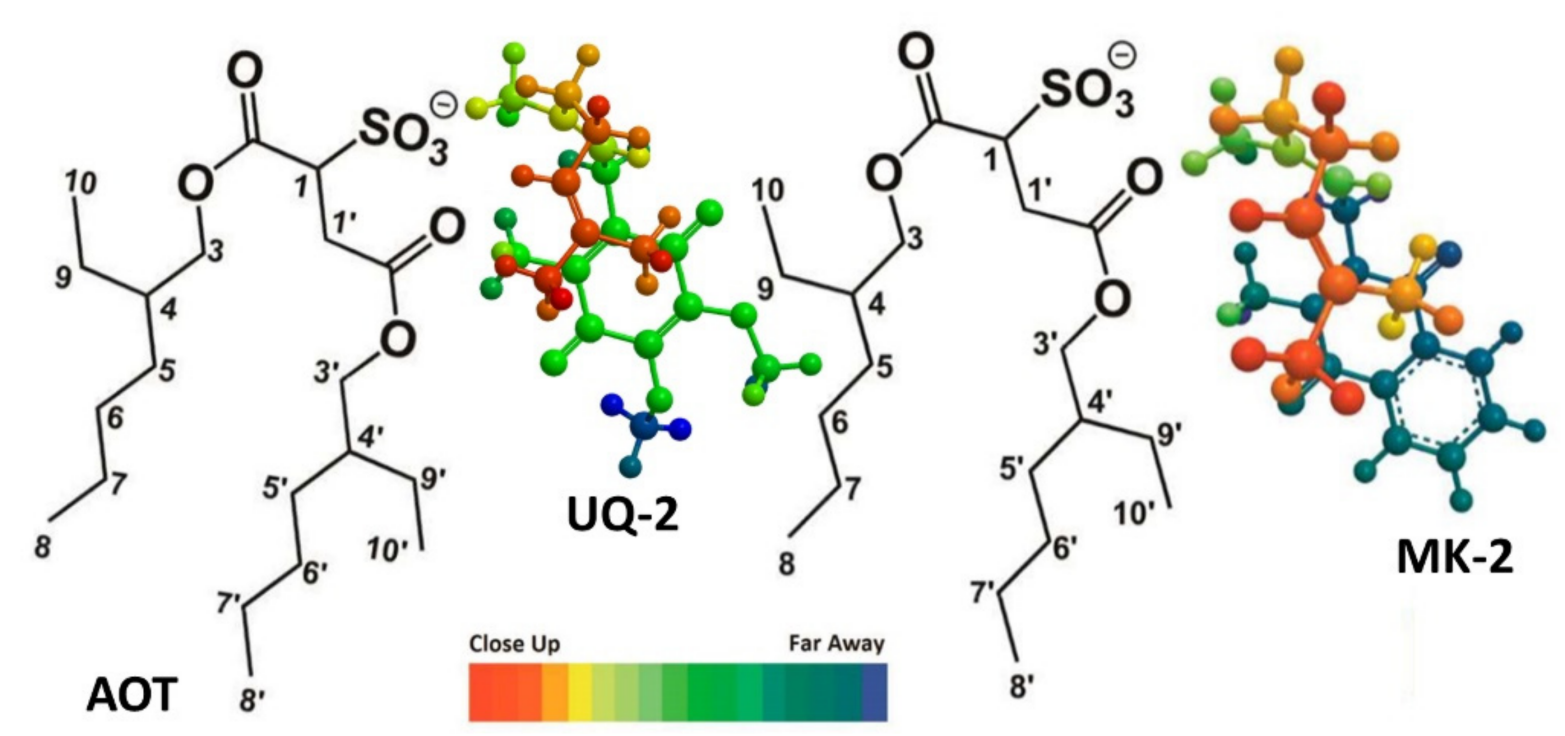
Comparison of the location of UQ-2 and MK-2 in AOT reverse micelles. AOT protons labeled provided. The color spectrum indicated relative depth when viewed from above. This figure was originally published in Ref. [41] and is licensed under CC-BY 4.0.

In a recent study by our group, truncated UQ-derivative, UQ-2, was found to be closer to the bulk water of the AOT reverse micelle model system than MK-2 using 2D ^1^H-^1^H NOESY and ROESY NMR spectroscopic methods [41]. The resulting intermolecular cross peaks between the lipoquinone and AOT led us to determine the location of each compound in RMs. Both lipoquinone derivatives were found near the interface; however, UQ-2 was slightly closer to the bulk water than MK-2 (Figure 13). This was determined by evaluating the cross peaks between the protons on the headgroups and the protons of AOT. Cross peaks between the methoxy protons of UQ-2 and H1-H4 of AOT (labeled in Figure 13) where absent from the MK-2 spectrum suggesting the UQ-2 headgroup is closer to the interface than MK-2 [60]. The deeper penetration of MK-2 in the interface could be attributed to the relative polarity of each headgroup, and the trend suggests PQ-2 could be found somewhere in between UQ-2 and MK-2, but closer to MK-2. This experimental study supports the computational work by Arantes and workers described above [1], and together strengthens the argument that the type of quinone headgroup has more influence on the location of the quinone and quinol groups in the membrane.

## 4. Synthesis of Lipoquinones

**Figure 14:**
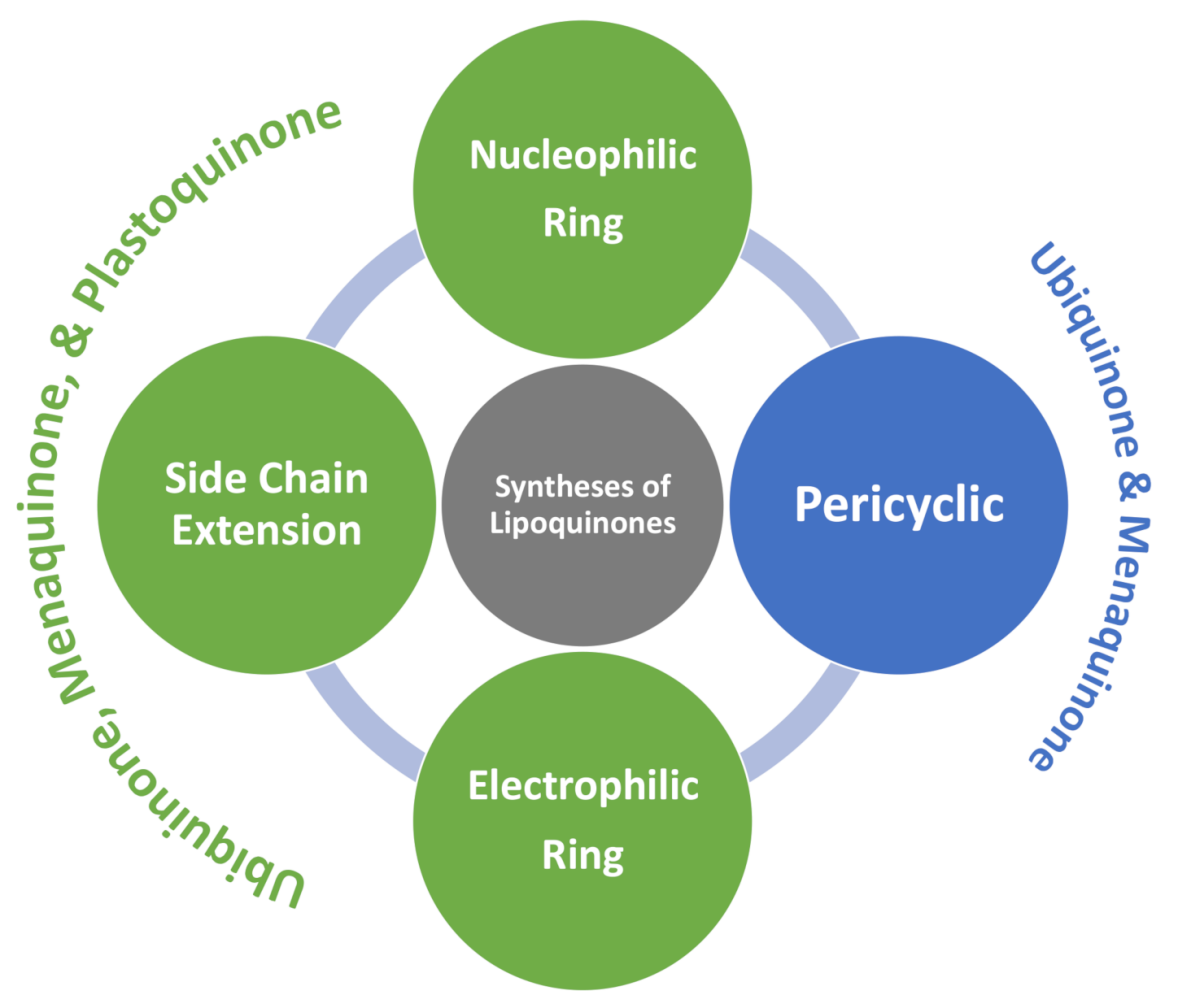
Synthetic methods used to synthesize lipoquinones.

Given the diversity in structure, only the most common lipoquinone derivatives are commercially available, and many of them must be prepared by the research group, synthetic collaborators, or acquired through custom synthesis. Here, we will describe an overview of the common approaches currently available (Figure 14) to make lipoquinone derivatives in reasonable amounts that can be used for biological studies. Some of the synthetic methods describes are feasible to the non-expert, so we provide a section here summarizing the available synthetic approaches.

The synthesis of all three lipoquinones have been achieved using nucleophilic ring methods, sidechain extensions, and electrophilic ring methods. Ubiquinones and menaquinones have also been synthesized using pericyclic methods. The number of steps, level selectivity, and technical skills required varies from method to method; however, each method yields product suitable for biological studies.

### 4.1. Nucleophilic Ring

**Scheme 3:**
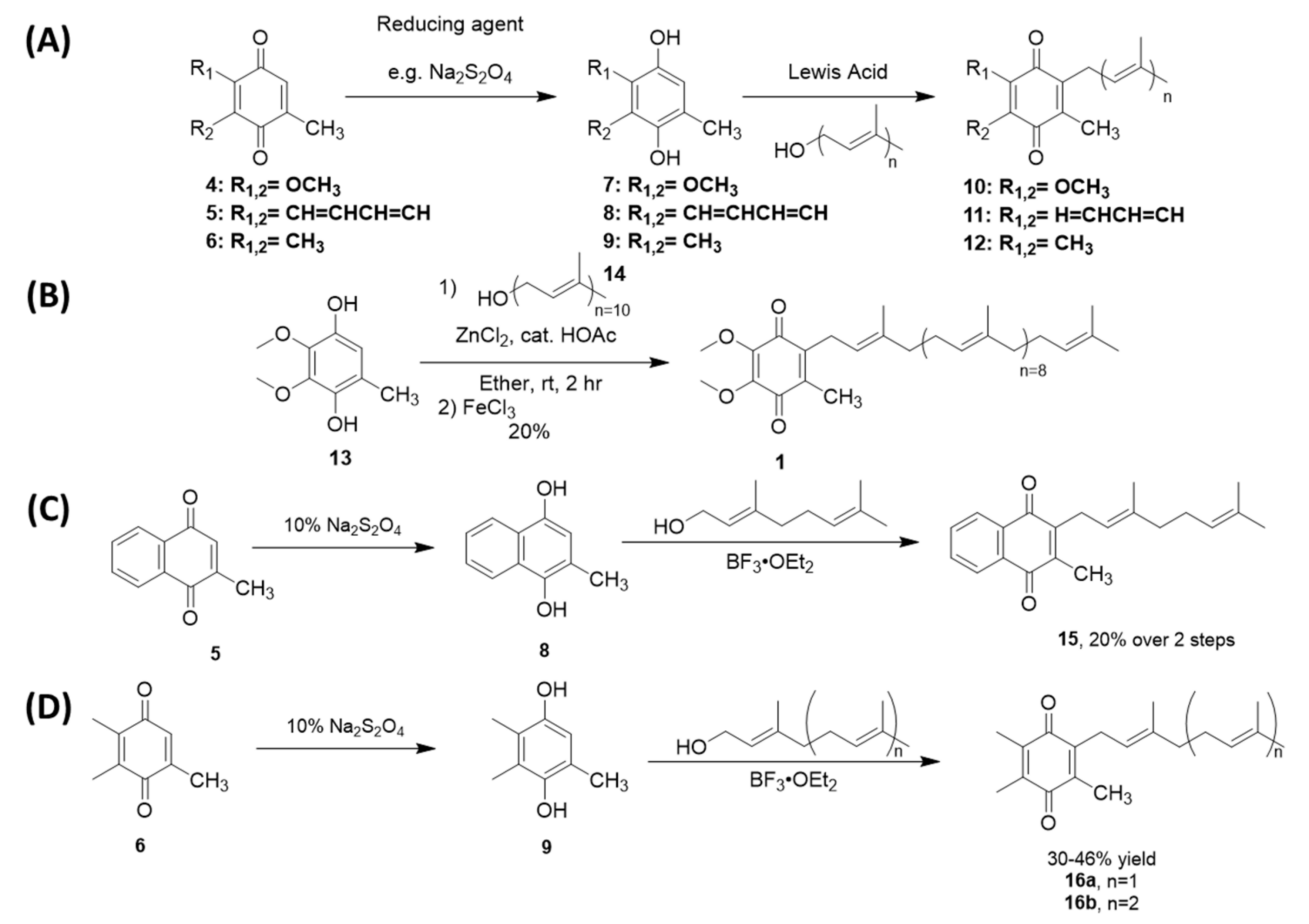
Examples of Friedel-Crafts reactions used to synthesize lipoquinones. (**A**). General reaction scheme for each lipoquinone. (**B**). Synthesis of UQ-10 [117]. (**C**). Synthesis of MK-2 [60,118]. (**D**). Synthesis of PQ-2 and PQ-3 [119].

Friedel-Crafts alkylation is one of the most common methods used to synthesize UQ-, MK-, and PQ-derivatives (Scheme 3). These methods are attractive for their 2–3 step reaction sequences. Each synthesis begins with the reduction of the quinone rings **4–6** to the corresponding quinol **7–9** in the presence of a reducing agent, such as sodium dithionite (Scheme 3A). The resulting quinol then undergoes Friedel-Crafts alkylation in the presence of a Lewis acid. For example, ZnCl_2_ was used in the conversion of ubiquinol **13** to UQ-10(H_2_) (not pictured), which was then further oxidized in the presence of FeCl_3_ to form **1** (Scheme 3B) [117]. BF_3_-OEt_2_ is the most popular Lewis acid for this purpose, which is conveniently used under inert atmosphere conditions. For example, it was used to synthesize MK-2 (**15**), a truncated derivative of MK-9, by Koehn and coworkers (Scheme 3C). First, menadione **5** was reduced form the quinol **8**. The Friedel-Crafts reaction was performed in the presence of BF_3_-OEt_2_, which was first reported by Suhara and coworkers [118], and commercially available geraniol to form **15** in 20% yield over two steps (Scheme 3A) [60,118]. It is important to note this route is impacted by the E/Z isomerism of the first isoprene unit leading to a low yield of the desired product. The reported characterization of MK-2 by Suhara and coworkers is also not very thorough or even absent, making verification of the product difficult. Proper separation and characterization of each isomer is important when moving forward with biological studies. Both PQ-2 (**16a**) and PQ-3 (**16b**) were synthesized using the same conditions (Scheme 3D) [119]. Although the yields are generally considered low, this synthesis is short, yields milligram quantities, and is attractive to prepare lipoquinones for chemical biology studies. The Friedel-Crafts approach is most often utilized by medicinal chemists and biologists because it is convenient and produces adequate amounts of product for biological study

### 4.2. Sidechain Extentions

**Scheme 4:**
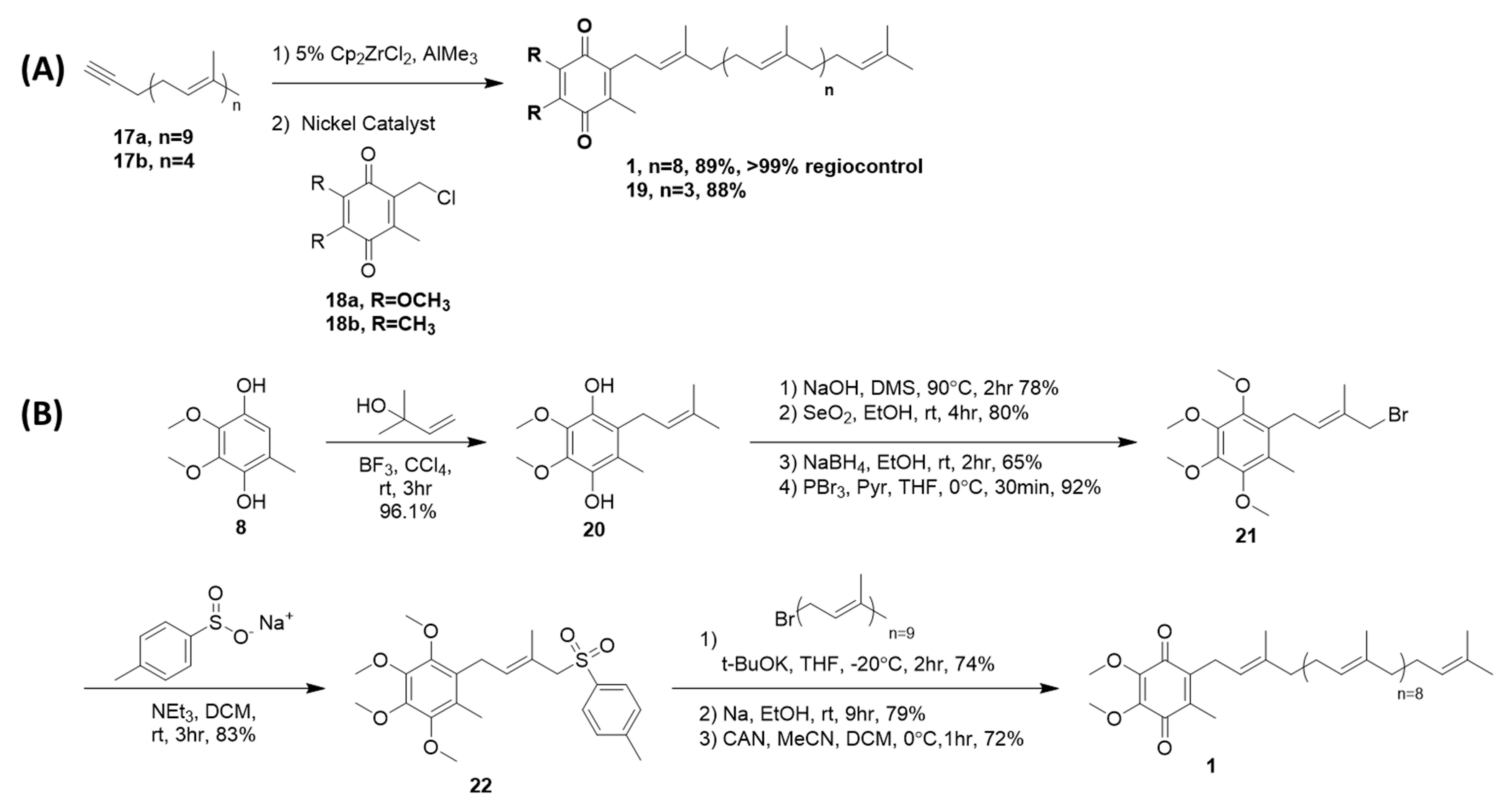
Examples of sidechain extension methods used to synthesize lipoquinones. (**A**) Synthesis of UQ-10 and PQ-5 through the elongation of the sidechain using Negishi carboalumination [121,124]. (**B**) Synthesis of UQ-10 using the sulfonation of the prenyl group and nucleophilic attack of the polyprenyl halide [123].

Sidechain extension methods are another common strategy to synthesize long-chain lipoquinone derivatives, such as UQ-10. The syntheses begin with the installation of a carbon via homologation of the headgroup or installation of a single prenyl group. From here, the sidechain is functionalized further to add the remaining length in segments or all at once (Scheme 4). For example, Lipshutz and coworkers utilized Negishi carboalumination to stereoselectively install the sidechain. Beginning with a polyprenyl terminal alkyne **17** (Scheme 4A), the sidechain is then installed to a chloromethylated quinone ring **18** in the presence of a nickel catalyst, like Ni^0^ or NiCl_2_(PPh_3_) [120,121,122]. Next, the elongation of the sidechain can also be achieved via the functionalization of the prenyl group. For example, Ravada and coworkers synthesized UQ-10 by reverse prenylation of **8** using Friedel-Crafts conditions to form quinol **20** [123]. One of the geminal dimethyl groups is transformed into an allyl bromide **21** via a four step sequence (Scheme 4B). The bromo group then undergoes substitution with sodium p-toluenesulfonate to form the tolyl sulfone **22**. The carbon alpha to the sulfone is easily deprotonated to form a nucleophile to undergo substitution with the electrophilic polyprenyl bromide. Removal of the sulfone occurs in the presence of dissolved sodium metal, resulting in the desired product **1**.

The literature shows no preference for using one method over the other, but it is important to note that both routes utilize very reactive reagents, such as organoaluminum and dissolved sodium metal, which requires significant technical skills to handle safely. However, Lipshutz and coworkers achieved coupling the headgroup to the sidechain in a convergent 2 step synthesis with excellent stereoselectivity and high yields [120,121,122,123].

### 4.3. Electrophilic Ring

**Scheme 5:**
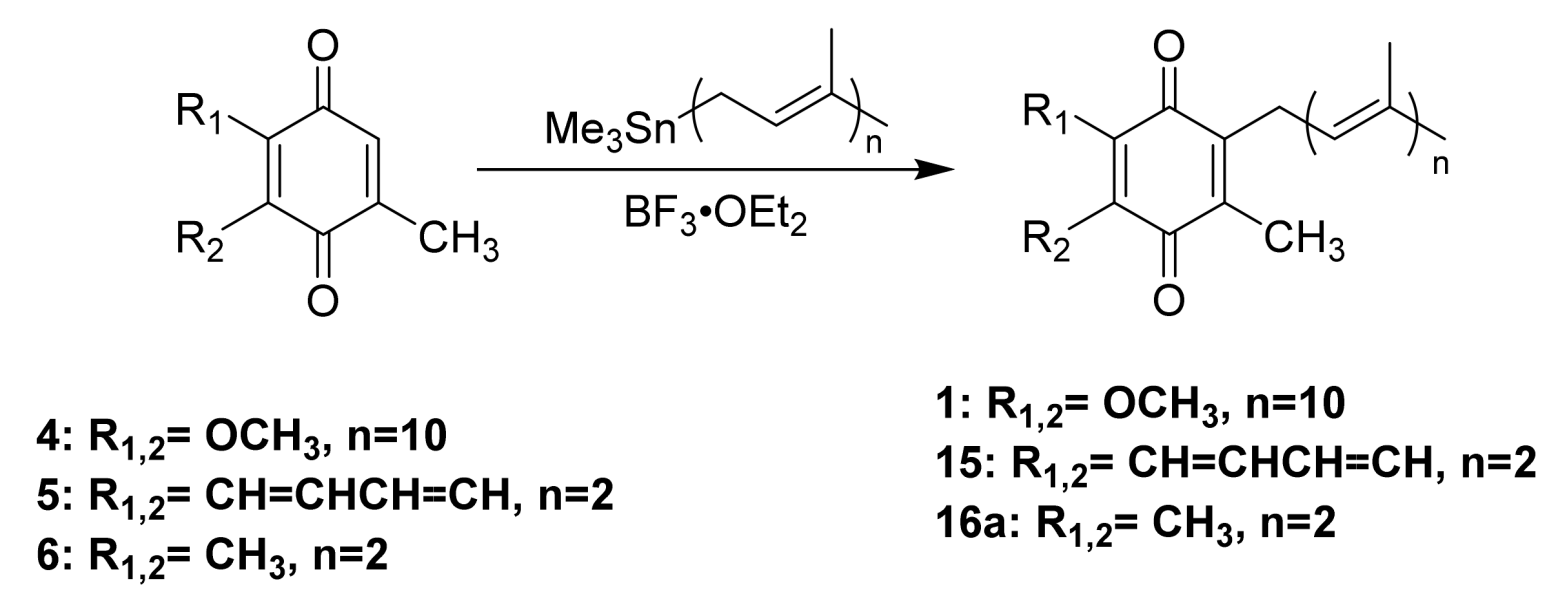
Examples of electrophilic ring methods used to synthesize lipoquinones using organostannane nucleophiles resulting in 1,2- and 1,4-additons to the ring Refs. [125,126].

UQ-, MK-, and PQ-derivatives have been synthesized in one step using organostannane reagents, specifically, UQ-10 (**1**), MK-2 (**15**), and PQ-2 (**16a**) (Scheme 5). Naruta and coworkers synthesized two geometric isomers of MK-2: MK-2 (with an *E* (or trans) alkene within the first isoprene unit) and MK-2(z1) (with a *Z* (or cis) alkene within the first isoprene unit). The stereochemistry of each MK-derivative was achieved using geranyl and neryl stannanes, respectively. To their surprise, they observed complete stereoretention of the alpha isoprene unit for each derivative. [125,126,127]. Therefore, the use of organostannane reagents could be one way to ensure the retention of stereochemistry at the racemizable double bond. Although their affinity for stereoretention of alkenes is attractive, the organostannane reagents have been observed to add via both 1,2- and 1,4-additions to the quinone, resulting in a mixture of products and requiring elaborate purification procedures to obtain product of sufficient purity for use in biological studies.

### 4.4. Pericylcic

**Scheme 6:**
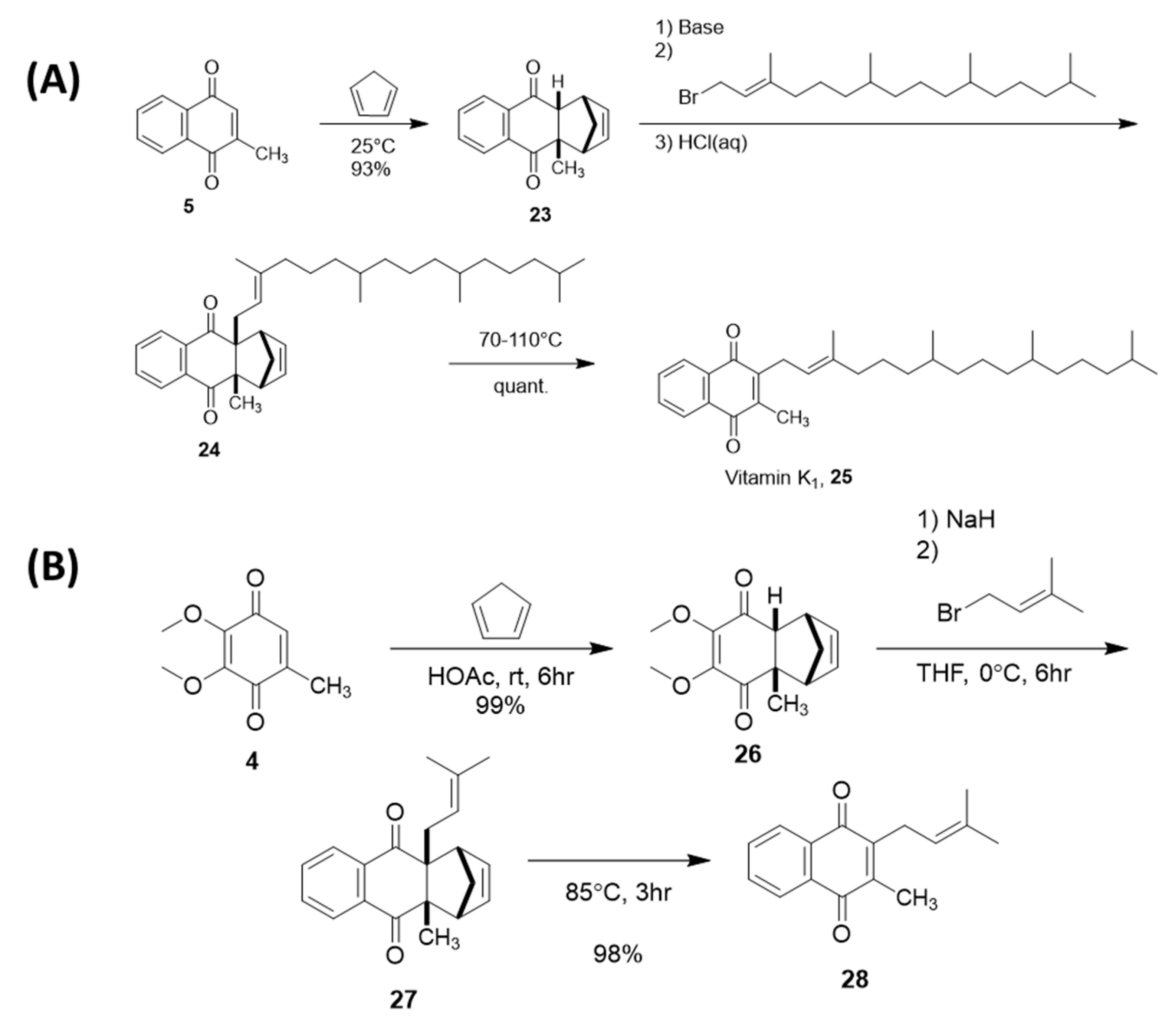
Examples of Diels−Alder reactions being used to control the regioselectivity of the sidechain installation. (**A**) Synthesis of vitamin K1 [128,129]. (**B**) Synthesis of UQ-1 [130].

Diels-Alder reactions have been used to synthesize various menaquinone and ubiquinone derivatives (Scheme 6) [128,129,130]. Application of the Diels-Alder reaction for preparation of lipoquinones is unquestionably the most elegant method. This regioselective approach takes advantage of the adduct formed between the quinone headgroup and cyclopentadiene, which results in a sp^3^ hybridized C2 and C3 carbons alpha to the carbonyls. In general, the C3 alpha proton is susceptible to deprotonation with a strong base to form the corresponding enolate. Alkylation can then occur with the respective prenyl halide to form the alkylated adduct. The alkylated adduct is unstable leading it to undergo spontaneous retro Diels-Alder reactions, but this is often facilitated through reflux.

This method gained popularity through the patented syntheses of vitamin K_1_ **25** on the gram scale (Scheme 6A) [128,129]. The cycloadduct **23** is formed between menadione **5** and cyclopentadiene. Enolate alkylation with phytyl bromide forms the functionalized adduct **24**. Vitamin K_1_, **25**, is formed via the subsequent retro-Diels-Alder reaction, removing the auxiliary cyclopentadiene group. A truncated derivative of ubiquinone, UQ-1 (**28**), has also been synthesized using the same approach via the cycloadduct **26** and alkylated-adduct **27** (Scheme 6B) [130]. Although conceptionally attractive, this approach has been carefully optimized for the reported syntheses, structure, and scale. This method introduces considerable competition between the enolate alkylation of the sidechain to the headgroup and an elimination reaction between the base and the prenyl halide. Therefore, more technical skill is required to achieve high selectivity, and it may not be as generally adoptable as desired to modified lipoquinone systems.

## 5. Summary and Future Outlook

Lipoquinone are hydrophobic molecules for which a major function is transport of electrons between membrane-bound proteins in the electron transport chain in cellular membranes. Understanding the location of lipoquinones in the membrane will have great impacts on experiments related to the use of such compounds in the fields of chemistry, biology, biochemistry, and medicine. Studies of more complex, protein-containing systems with established baseline locations of lipoquinones in such membrane environments would allow observation of how their locations change in the presence of proteins. This would have implications in the emerging “swimming” vs. “diving” arguments for lipoquinone location, which are largely supported by computational studies. However, the understanding of the cellular environment has changed, and these environments are more crowded and consequently less polar than previously believed, thus supporting the conclusions regarding the existence of concentrations of “swimming” vs. “diving“ lipoquinones. A baseline location would also help clarify the behavior and function of the lipoquinone pool, which is thought to be within the midplane between membrane-bound protein complexes of the electron transport chain and surface of the membrane; however, as we show above, that depends on the length of the sidechain. However, as we also described a Coq10 chaperone protein whose function it is to help Q exert its action. Hence the notion that the location is only determined by the properties of the lipoquinone and the membrane is a simplification. On the other hand, it is unlikely that the protein binding Q is the only determinant in the location of the lipoquinone considering that there are more molecules of lipoquineones than binding proteins. The location of lipoquinones remains important since some lipoquinones are likely bound to their potential chaperone proteins, whereas others form a pool which serves as a reservoir to replenish the quinone electron donor and acceptor function in the membrane.

The location of the lipoquinone in the membrane will also define their local chemical environments with more polar or nonpolar regions. The chemical environment alters the conformation of the lipoquinones and changes of the electrochemical potential will depend on both the lipoquinone’s location and wether it is bound to a protein or not. Structure and location thus remain useful parameters when considering the electrochemical properties of the lipoquinone in specific environments. The analysis and general findings could potentially be extended to other isoprenoid and olefinic biomolecules, although the specific details will depend on the structure, location, environment, and specific function of the molecule.

Although simple in construction, lipoquinones are deceptively complex molecules. Their physical properties are often ignored or misinterpreted, leading to conflicting conclusions about their behavior and function in the literature. Their roles as electron transport agents in association with proteins in the electron transport chain have been widely studied, but little is known regarding their behavior within the membrane. For example, studies looking into the location of the quinone headgroup are riddled with contradictory reports. Often studies have been performed with a variety of different experimental and computational approaches, model membrane systems, and analytical methods, which make it difficult to directly compare the results. When discussing such studies, it is important to remember lipoquinones are very hydrophobic and hence it is difficult to ensure compound dissolution in aqueous-containing environments. To overcome this, many researchers have turned to computational methods to investigate the location and conformation of lipoquinones in solvent and membrane environments, which does not require consideration of solubility. Similarly, the redox potentials of lipoquinones are sensitive to local environments, and therefore their function is likely to be closely tied to their location, although at this point, limited information is available on this topic. Many studies are limited because the programs, datasets, and parameters used to describe the properties are not uniform; therefore, the resulting interpretations and conclusions may vary significantly from study to study.

An attempt to overcome some of these limitations has recently been introduced in an experimental approach using truncated lipoquinone derivatives to override solubility problems. Although still hydrophobic, short-chain lipoquinones derivatives, such as UQ-2 and MK-2, are more soluble in both organic and aqueous media, making them perfect candidates for comparison studies. Our group has demonstrated their usefulness and evaluated the locations of short chain UQ and MK in AOT reverse micelles using 1D and 2D NMR spectroscopy and in Langmuir monolayers trough studies. Our results support the importance of the headgroup on lipoquinone location within the membrane, and this conclusion was supported by Arantes computational work with similar derivatives with varying sidechain lengths. As such we have clarified the ongoing controversy on the location of the menaquinones in the membrane.

In order to carry out any studies, the compounds must be available, and hence we summarize several approaches reported to synthesize lipoquinones when they are not commercially available. Reported methods will assist the experimentalist interested in using modified or isotopically labeled lipoquinone derivatives and can facilitate investigations of biological function. Lipoquinone derivatives are accessible, and structural modifications could assist future studies into exploring the fundamental properties of the biological systems or potential applications of systems requiring lipoquinones.

We emphasize the importance for researchers in the future to bridge the gap between experimental and computational methods and incorporate properties-focused, experimental studies to support computational findings. As the field moves forward, considerations of more realistic biomimetic membranes (including lipids, cholesterols, and proteins) will be paramount to understanding the true behavior of lipoquinones in the membrane, particularly if the location and conformation of lipoquinones in the membrane and possible interactions with Coq10 chaperone protein are important to the questions being addressed.

## Figures and Tables

**Table 1:** Summary of references regarding the membrane location of the quinone headgroup in lipoquinones. Experimental studies will be noted with superscripted “^E^”, and computational studies will be denoted with superscripted “^C^”.

Lipoquinone	Near Lipid Headgroups	Within Acyl Chains	Within Midplane
Ubiquinone	Kingsley and Feigenson ^E^ [33]	Michaelis and Moore ^E^ [42]	Ulrich et al. ^E^ [49]
	Stidham et al. ^E^ [34]	Cornell et al.^E^ [43]	Ondarroa and Quinn ^E^ [50]
	Lenaz et al.^E^ [35]	Chazotte et al.^E^ [44]	Soderhall and Laaksonen ^C^ [51]
	Galassi and Arantes ^C^ [36]	Metz et al.^E^ [45]	
	Gómez-Murcia et al. ^E^ [37]	Afri et al. ^E^ [46]	
	Kaurola et al. ^C^ [38]	Hauss et al. ^E^ [47]	
	Quirk et al. ^E^ [39]	Salgado et al. ^E^ [48]	
	Teixeira and Arantes ^C^ [40]		
	Braasch-Turi et al. ^E^ [41]		
	Salgado et al. ^E^ [48]		
Plastoquinone			Millner and Barber [54]
	de Jong et al. ^C^ [52]		Joliot et al. ^E^ [55]
	van Eerden et al. ^C^ [53]		Marchal et al. ^E^ [56]
			Kirchoff et al. ^E^ [57]
			Loll et al. ^C^ [58]
			Lambreva et al. ^C^ [59]
Menaquinone	Koehn et al. ^E^ [60] Van Cleave et al. ^E & C^ [1]		

**Table 2:** LogP and clogP values of select lipoquinones from the literature.

LogP Values				
Lipoquinone	PubChem	Molinspiration	Chemicalize	Experimental
UQ-0	0.8	0.22	0.608	0.78 ^a^
UQH_2_-0	1	1.62	1.564	1.02 ^a^
UQ-1	2.8	2.69	2.219	>3 ^a^
UQH_2_-1	3.2	3.63	3.293	>3 ^a^
UQ-2	4.6	4.53	3.878	
UQH_2_-2	5.3	5.47	4.952	
UQ-4	8.3	8.2	7.198	8.3 ^b^
UQ-6	12	9.59	10.517	12 ^b^
UQ-8	15.7	10.15	13.837	15.7 ^b^
UQ-10	19.4	10.51	17.156	19.4 ^b^, 14.72 ^c^
UQH_2_-1	20.2	10.58	18.23	12.26 ^c^
PQ-0	1.2	1.31	1.82	
PQH_2_-0	1.2	2.67	2.39	
PQ-1	3.4	3.09	3.43	>3 ^a^
PQH_2_-1	3.3	4.24	4.12	>3 ^a^
PQ-2	5.2	4.94	5.086	
PQH_2_-2		6.09	5.781	
PQ-9	18.2	10.38	16.704	
PQH_2_-9	18.1	10.48	17.399	
MK-0	2.2	2.04	1.89	2.1 ^a^
MKH_2_-0	2.8	2.99	2.869	
MK-1	3.4	3.83	3.501	3.474 ^d^
MKH_2_-1	4.8	4.77	4.598	1.36 ^a^
MK-2	5.2	5.67	5.16	4.952 ^d^
MKH_2_-2	6.6	6.62	6.257	
MK-3	7.1	7.52	6.82	6.43 ^d^
MK-4	8.9	8.86	8.48	7.909 ^d^
MK-5	10.8	9.44	10.139	9.387 ^d^
MK-6	12.6	9.8	11.799	10.866 ^d^
MK-7	14.5	10.7	13.459	12.344 ^d^
MK-8	16.4	10.28	15.119	
MK-9	18.2	10.45	16.778	
MKH_2_-9	19.6	10.53	17.875	

Experimental logP values from Rich and Harper, 1990 [61] ^a^, Fedor et al., 2017 [62] ^b^, Tekin and Erkoc, 2010 [63] ^c^, Ishihara and Sakagami, 2007 [64] ^d^.

**Table 3:** Summary of the studies reported relating to the location of the ubiquinone headgroup.

Author(s)	Compound(s)	Method(s)	Type of Membrane	Location of Quinone Headgroup	Location of Sidechain
Kingsley and Feigenson, 1981 [33] *	UQ-1-10 & UQ(H_2_)-1-10	^1^H NMR with chiral shift (Dy^3+^ & Tm^3+^)	DMPC-*d*72	Polar headgroups of lipids	Increase number of isoprenes, further into acyl tails
Stidham et al., 1984 [34] *	UQ-10	^13^C NMR, light microscopy, & XRD	DMPC, DPPC, & EPC	Polar headgroups of lipids	Increase number of isoprenes, further into acyl tails
Ulrich et al., 1985 [49] *	UQ-2, 3, & 10	UV-Vis & NMR. Measured the rate of reduction of UQ with NaBH_4_ & dithionite	DMPC & DPPC	Deep enough in acyl tails to not to be reduced by NaBH_4_	UQ-2 and 3, close to interface, UQ-10 deep in membrane
Michaelis and Moore, 1985 [42] *	UQ-10	^1^H NMR with chiral shift (Pr^3+^ & Eu^3+^) & Electron microscopy	EPC	Acyl chains at least beyond C2	
Ondarroa and Quinn, 1986 [50] *	UQ-10	^1^H NMR with chiral shift (Dy^3+^) & ^31^P NMR	DPPC & EPC	Midplane	
Cornell et al., 1987 [43] *	UQ-10 & various deuterated derivatives	^1^H NMR, solid state ^2^H NMR with deuterated lipids, & enzyme assays	dispersed in *E. Coli* plasma membranes & inner membranes of beet root mitochondria, DMPC & EPC	Acyl chains to midplane	
Lenaz et al., 1992 [35] *	UQ-3 & UQ-10	fluorescence quenching & linear dichroism	phospholipid purified from asolectin	UQ-3 is found throughout, UQ-10 was found to be parallel to acyl chains, headgroup near interface	
Chazotte et al., 1991 [44] ^†^	NBDHA-Q (Fluorescent UQ-Derivative)	Fluorescence recovery after photobleaching & fluorescence spectroscopy	DMPC with & without cholesterol	Acyl chains to midplane. “Cholesterol region”	Isoprene tail by interface likely due to the fluorescent linker
Salgado et al., 1993 [48] *	UQ-10 & UQ-10(H_2_)	magic angle ^13^C NMR with Gd^3+^ chiral shift	Egg yolk phosphatidylcholine	Quinol perturbs membrane surface more than quinone- suggesting quinone is closer to the tails.	
Metz et al., 1995 [45] *	^13^C-UQ-10	^13^C NMR with Gd^3+^ chiral shift	DMPC & DHPC	headgroup buried in the lipid bilayer or in the midplane. Headgroup preferentially parallel to lipid chains	
Soderhall and Laaksonen, 2001 [51] ^‡^	UQ-et & UQ-10	nanosecond MD calculations	DPPC bilayer	UQ-et between C4-C10 of palmitic tails, UQ-10, 2 positions: one close to lipid headgroup other close to midplane	UQ-10 able to move with headgroup and aligned parallel to palmitic tails
Afri et al., 2004 [46] *	UQ-10 & UQ-10(H_2_)	NMR chemical shift-polarity correlation, 1D H and C, 2D COSY, HMQC, HMBC, and NOESY, measured rate of reduction of substrates with vitamin C	DMPC vesicle	Deep enough in acyl tails not to be reduced by vitamin C	Embedded in “slab”
Hauss et al., 2005 [47] *	UQ-10	neutron diffraction	deuterated DMPC doped with deuterated DMP-serine	Midplane	In midplane, may not be extended, could be “curled”
Galassi and Arantes, 2015 [36] ^‡^	UQ-0,1,2 & 6 UQ(H_2_)-0,1,2 & 6	MD calculations	POPC	Water-bilayer interface near glycerol groups, perpendicular to midplane, does not change significantly with length of tail	In acyl tails, moves with headgroup
Gómez-Murcia et al., 2016 [37] ^†^	idebenone & idebenol	differential scanning calorimetry, X-ray diffractions, and ^2^H NMR and NOESY MAS	DPPC, POPC, and deuterated DPPC	Close to lipid-water interface by NOESY	
Kaurola et al., 2016 [38] ^‡^	UQ & UQ(H_2_) 1,6,8,9,10	atomistic MD calcs	DLPC, DLPE, CL	Acyl groups right under headgroup region, stabilizes in midplane	
Quirk et al., 2016 [39] *	UQ-10	Surface-enhanced infrared adsorption spectroscopy & neutron reflectometry	deuterated DMPC	IR vibrations suggest headgroup is in both polar and nonpolar environments	Likely in midplane and has freedom to move with headgroup
Teixeira and Arantes, 2019 [40] ^‡^	UQ-2,10 & UQ(H_2_)-1,10, MK-9	MD calculations	POPC, DLPE, DLPC	Polar headgroups of lipids	
Murai M et al., 2014, [103] Allan et al., 2015 [104] Tsui et al., 2019 [105]	UQ-derivatives (Coq10 and Coq11)	Genetic and biochemical studies including photoaffinity labeling	*Saccharomyces cerevisiae*	Associated with Coq10 in the hydrophobic channel	Associated with Coq10 in the hydrophobic channel
Murai M et al. [103] Tsui et al., 2019 [105] Hernandez-Camacho 2018, [106]	UQ-derivatives	Cellular studies (including genetics, and animal studies), X-ray and docking calculations	Human mitochondrial membrane	Associated with Coq10 in the hydrophobic channel	Associated with Coq10 in the hydrophobic channel

* Samples prepared using similar procedures: dissolve analyte and lipids in polar solvent, remove solvent, and rehydrate in D_2_O. ^†^ Modified ubiquinone analyte. ^‡^ Computational study. Type of membrane abbreviations definitions: dimyristoylphosphatidylcholine (DMPC), dipalmitoylphosphatidylcholine (DPPC), egg phosphatidylcholine (EPC), diheptanoylphosphatidylcholine (DHPC), palmitoyloleoylphosphatidylcholine (POPC), dilinoleoylphosphatidylcholine (DLPC), dilauroylphosphoethanolamine (DLPE), and cardiolipin (CL).

## Data Availability

This is a review. Data can be found in the cited papers. Additional contact should be made to the respective corresponding authors or to Debbie C. Crans (debbie.crans@colostate.edu).

## Abbreviations

AOT	bis(2-ethylhexyl)sulfosuccinate sodium
AOT RMs	bis(2-ethylhexyl)sulfosuccinate sodium reverse micelles
ATP	adenosine triphosphate
CG	Coarse Grain
CL	cardiolipin
CV	cyclic voltammogram
DLPC	dilinoleoylphosphatidylcholine
DLPE	dilauroylphosphoethanolamine
DMPC	dimyristoylphosphatidylcholine
DMSO	dimethyl sulfoxide
DPPC	dipalmitoylphosphatidylcholine
EPC	egg phosphatidylcholine
MD	Molecular Dynamics
MeCN	acetonitrile
MK	menaquinone
NBDHA	6-(N-(7-nitrobenz-2-oxa-1,3-diazol-4-yl)amino)hexanoic acid
NMR	nuclear magnetic resonance
NOESY	Nuclear Overhauser Effect Spectroscopy
PBD	Protein Data Bank
POPC	palmitoyloleoylphosphatidylcholine
PQ	plastoquinone
ROESY	Rotating Frame Overhauser Enhancement Spectroscopy
UA	United Atom
UQ	ubiquinone
UV	ultraviolet
XRD	X-Ray Diffraction
